# A subset of topologically associating domains fold into mesoscale core-periphery networks

**DOI:** 10.1038/s41598-019-45457-9

**Published:** 2019-07-02

**Authors:** Harvey Huang, Sunnia T. Chen, Katelyn R. Titus, Daniel J. Emerson, Danielle S. Bassett, Jennifer E. Phillips-Cremins

**Affiliations:** 1 Department of Bioengineering, School of Engineering and Applied Sciences, University of Pennsylvania, Philadelphia, PA 19104 USA; 2 Epigenetics Institute, Perelman School of Medicine, University of Pennsylvania, Philadelphia, PA 19104 USA; 3 Department of Genetics, University of Pennsylvania, Philadelphia, PA 19104 USA; 4 Department of Electrical & Systems Engineering, School of Engineering and Applied Sciences, University of Pennsylvania, Philadelphia, PA 19104 USA; 5 Department of Neurology, Perelman School of Medicine, University of Pennsylvania, Philadelphia, PA 19104 USA; 6 Department of Physics & Astronomy, College of Arts and Sciences, University of Pennsylvania, Philadelphia, PA 19104 USA

**Keywords:** Network topology, Epigenomics, Functional genomics, Regulatory networks

## Abstract

Mammalian genomes are folded into a hierarchy of compartments, topologically associating domains (TADs), subTADs, and long-range looping interactions. The higher-order folding patterns of chromatin contacts within TADs and how they localize to disease-associated single nucleotide variants (daSNVs) remains an open area of investigation. Here, we analyze high-resolution Hi-C data with graph theory to understand possible mesoscale network architecture within chromatin domains. We identify a subset of TADs exhibiting strong core-periphery mesoscale structure in embryonic stem cells, neural progenitor cells, and cortical neurons. Hyper-connected core nodes co-localize with genomic segments engaged in multiple looping interactions and enriched for occupancy of the architectural protein CCCTC binding protein (CTCF). CTCF knockdown and *in silico* deletion of CTCF-bound core nodes disrupts core-periphery structure, whereas *in silico* mutation of cell type-specific enhancer or gene nodes has a negligible effect. Importantly, neuropsychiatric daSNVs are significantly more likely to localize with TADs folded into core-periphery networks compared to domains devoid of such structure. Together, our results reveal that a subset of TADs encompasses looping interactions connected into a core-periphery mesoscale network. We hypothesize that daSNVs in the periphery of genome folding networks might preserve global nuclear architecture but cause local topological and functional disruptions contributing to human disease. By contrast, daSNVs co-localized with hyper-connected core nodes might cause severe topological and functional disruptions. Overall, these findings shed new light into the mesoscale network structure of fine scale genome folding within chromatin domains and its link to common genetic variants in human disease.

## Introduction

Seminal studies based on microscopy^1–3^ or molecular proximity ligation^4–8^ have revealed that chromatin is non-randomly folded in unique patterns at disparate length scales. Individual chromosomes segregate into independent territories with respect to each other^9^. Within each chromosome, active and inactive genomic regions partition into large-scale ‘A’ and ‘B’ compartments distinguished by activating and repressive chromatin modifications, respectively^5^. Within compartments, chromatin is further partitioned into Megabase (Mb)-sized topologically associating domains (TADs) and smaller, nested subTADs^6,10–14^. TADs/subTADs are demarcated by boundaries and have been formally defined as contiguous genomic intervals in which the majority of loci interact more frequently with each other than with loci outside of the domain^11,15^. At the sub-Mb scale, distal genomic segments can form specific physical contacts called looping interactions within and between subTADs^11,16–19^. The highest frequency loops in a population of cells are made manifest in Hi-C maps as punctate enriched peaks of contact between two genomic fragments compared to the surrounding TAD background signal. Finally, at the smallest length scale, DNA wraps around the histone octamer to form a 10 nm chromatin fiber. Understanding how chromatin architecture is connected to genome function is important because it sheds light on the molecular mechanisms governing healthy development and how these mechanisms go awry in disease.

Computational tools from network science and graph theory have been employed to characterize the structural features of real-world complex systems across a range of length scales^20,21^. Local and global properties of networks are straightforward to compute because the units of analysis, individual nodes and the whole network, are immediately evident and require no additional search. Mesoscale structure, however, is not always evident^22^. The difficulty largely lies in the fact that the identification of mesoscale structure requires the specification of a partition of a network’s nodes into groups (the units of analysis) based on a given criterion^23^. Real-world networks are composed of many nodes and edges arranged in complex patterns that can obscure structural regularities. Due to this complexity, if one wishes to identify mesoscale structure in networks, one must frequently search for it algorithmically^24^. Recently, algorithmic approaches explicitly developed for this purpose have uncovered assortative communities^25,26^, cores and peripheries^27^, and bipartite or disassortative structures^28^ in a wide range of diverse complex systems^29,30^.

We reasoned that an exploration of mesoscale network properties in high-resolution 3-D genome folding maps might lead to new understanding of the link between genome structure and function. Recently, our group and others have used network modularity maximization and the tuning of a resolution parameter, γ, to uncover a hierarchy of partially overlapping, nested communities in human Hi-C data genome-wide^31,32^. By smoothly tuning the resolution parameter from high to low extremes, we can effectively obtain estimates of a network’s community structure, spanning from the coarsest scale at which all network nodes fall into the same community to the finest scale where network nodes form singleton communities^32,33^. This so-called multi-scale community detection methodology ultimately revealed that nested communities in network science are synonymous with TADs/subTADs and have strong utility in their sensitive and specific detection genome-wide from Hi-C maps^31,32^.

Another feature of 3-D genome folding thought to fall in the range of mesoscale intermediate-length structure is the long-range loop^11^. We hypothesized that looping interactions within chromatin domains might form meso-scale network structures with importance for genome function. Here, we demonstrate that a subset of TADs genome-wide are folded into core-periphery networks, whereas size-matched, randomly positioned genomic intervals show no such structure. Core nodes directly correspond to genomic fragments engaged in multiple, highly connected looping interactions. Interconnected core nodes are strongly enriched for constitutively bound CTCF, whereas cell type-specific enhancers and genes are generally evenly distributed across both core and periphery nodes. Experimental knockdown of CTCF and *in silico* mutation of CTCF-occupied nodes disrupts core-periphery network structure, whereas *in silico* mutation of cell type specific enhancer or gene nodes has a negligible effect. Importantly, we discovered that common single nucleotide variants (SNVs) associated with key neuropsychiatric diseases, including autism spectrum disorder (ASD), schizophrenia, and obsessive-compulsive disorder (OCD), are strongly enriched in core-periphery TADs in cortical neurons. Together, these results reveal a subset of TADs that are folded in mesoscale core-periphery networks. We hypothesize a working model in which daSNVs that disrupt the highly interconnected core nodes might result in severe topological and functional disruption. By contrast, we posit that daSNVs in peripheral nodes might preserve global genome folding, but result in slight local topological and functional disruptions contributing to human disease.

## Results

**Figure 1:**
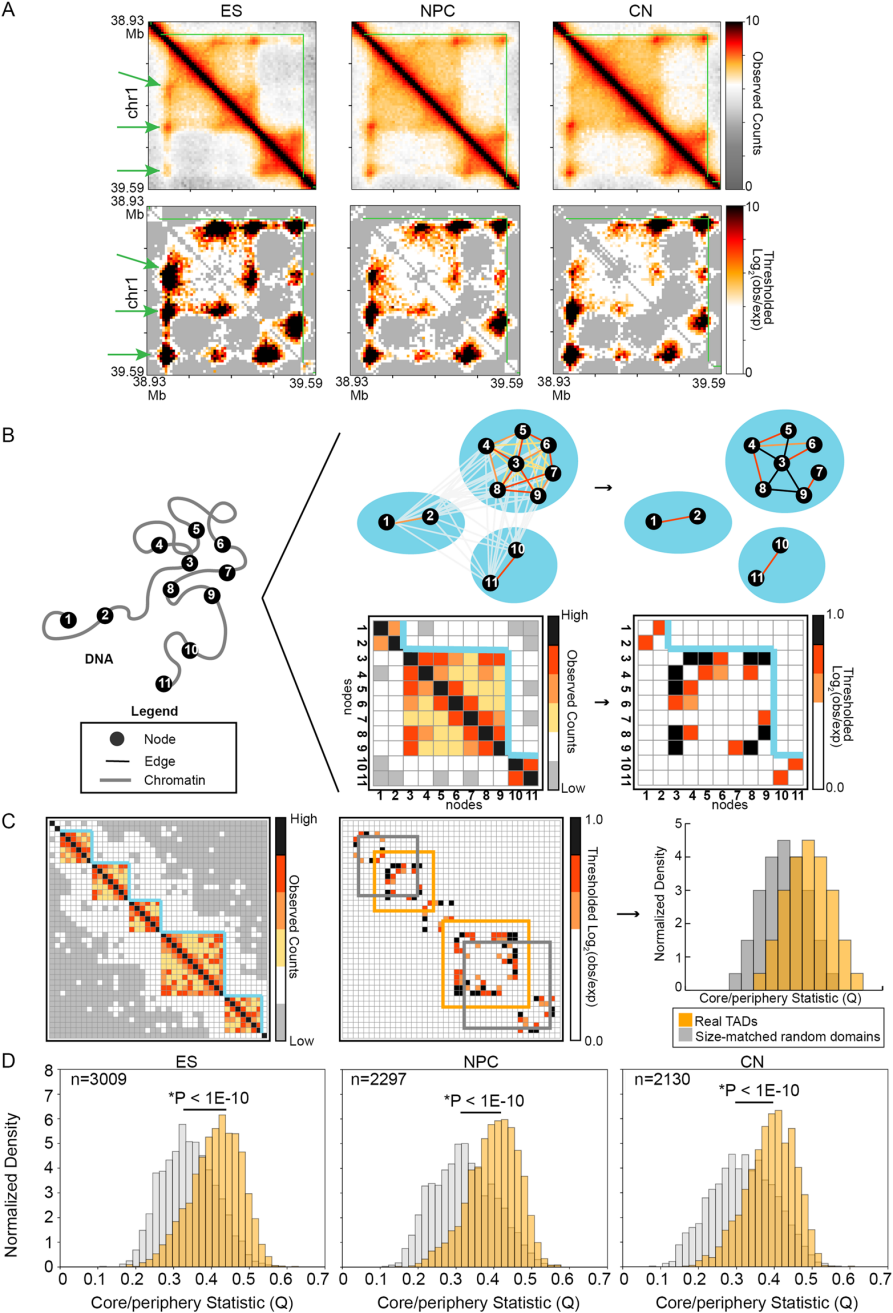
Genome folding within topologically associating domains (TADs) can exhibit  mesoscale core-periphery network structure. (**A**) Relative interaction frequency (Observed) and background-corrected interaction frequency (Observed/Expected) Hi-C heatmaps for a given TAD in mouse embryonic stem (ES) cells, neural progenitor cells (NPCs), and cortical neurons (CNs). Green lines, borders of TADs. Green arrowheads, genome structure signal indicative of a persistent looping interaction in ensemble Hi-C maps. (**B**) 3-D genome folding can be represented as a network where nodes correspond to bins of DNA and edges correspond to bin-bin interaction frequencies. Interaction frequency heatmaps of genome folding can be represented as adjacency matrices, allowing tools from graph theory and network science to be readily applied. Blue lines represent TAD boundaries. (**C**,**D**) To test for the possible presence of core-periphery structure created by looping interactions within TADs, we computed the core-periphery quality index Q for all true TADs compared to pseudo-TADs generated by choosing size-matched genomic regions at random. A Kolmogorov–Smirnov test was used to compare Q values for TADs vs. randomly placed, size-matched genomic regions in ES cells, NPCs, and CNs.

The mesoscale network patterns created by genome folding within TADs are poorly understood. Progress in exploring such a question has been hindered by the paucity of ultra-high resolution, genome-wide Hi-C maps across multiple mammalian cell types. Recently, Cavalli and colleagues published kilobase (kb)-resolution Hi-C maps with the highest read depth to date allowing for the genome-wide identification of looping interactions in mouse embryonic stem (ES) cells, primary neural progenitor cells (NPCs), and primary cortical neurons (CNs)^34^. We created interaction frequency maps at four kb resolution in all three cell types (Fig. 1A, Supplementary Methods, Supplementary Table 1). By applying the well-established directionality index and hidden Markov model methodology^6^, we identified TADs genome-wide in all three cell types (Supplementary Tables 2–7). We also modeled the distance-dependence and local TAD structure to compute an expected count for every pixel (Supplementary Figs 1–2, Supplementary Methods), thus enabling us to compute background-corrected interaction frequency (Observed/Expected) heatmaps (Fig. 1A). Thus, we created Observed/Expected heatmaps to enable the exploration of mesoscale network structure created by looping interactions within TADs genome-wide in mouse ES cells, NPCs, and CNs.

Visual inspection of the heatmaps revealed that many TADs contain bins that form multiple looping interactions (green arrows, Fig. 1A) with other highly interconnected nodes. This observation is reminiscent of core-periphery structure, a mesoscale organization of a graph that is often studied in network science. Core nodes exhibit high signal strength and are highly connected (high degree) to other highly connected nodes. By contrast, periphery nodes interact with core nodes but are rarely connected to each other. To explore the possibility of core-periphery network structure within TADs, we conceptualized Hi-C data as a network with each genomic bin represented as a node and Observed/Expected interaction frequencies between nodes represented as edges (Fig. 1B). We represented each Observed/Expected interaction frequency matrix for every TAD and cell type as an independent network (Fig. 1B). To quantify the strength of a TAD network’s core-periphery structure, we computed a core-periphery statistic, *Q*, using the Kernighan-Lin algorithm to optimize a quality index measuring core-periphery node separation (detailed in Supplementary Methods). In brief, we sought to maximize the interaction strength among core nodes and to minimize the interaction strength among periphery nodes. Intuitively, this quality index Q will be high when a TAD contains strong core-periphery network structure and low in TADs devoid of such structure. We observed that genome folding within TADs genome-wide in mouse ES cells, NPCs, and CNs exhibit significantly stronger core-periphery structure than expected in two null models: (i) pseudo-TADs where matched-size genomic intervals are positioned at random (Fig. 1C,D) and (ii) Erdős–Rényi random graphs (Supplementary Fig. 3). These results suggest that genome folding within at least a subset of TADs can exhibit mesoscale core-periphery network structure.

**Figure 2:**
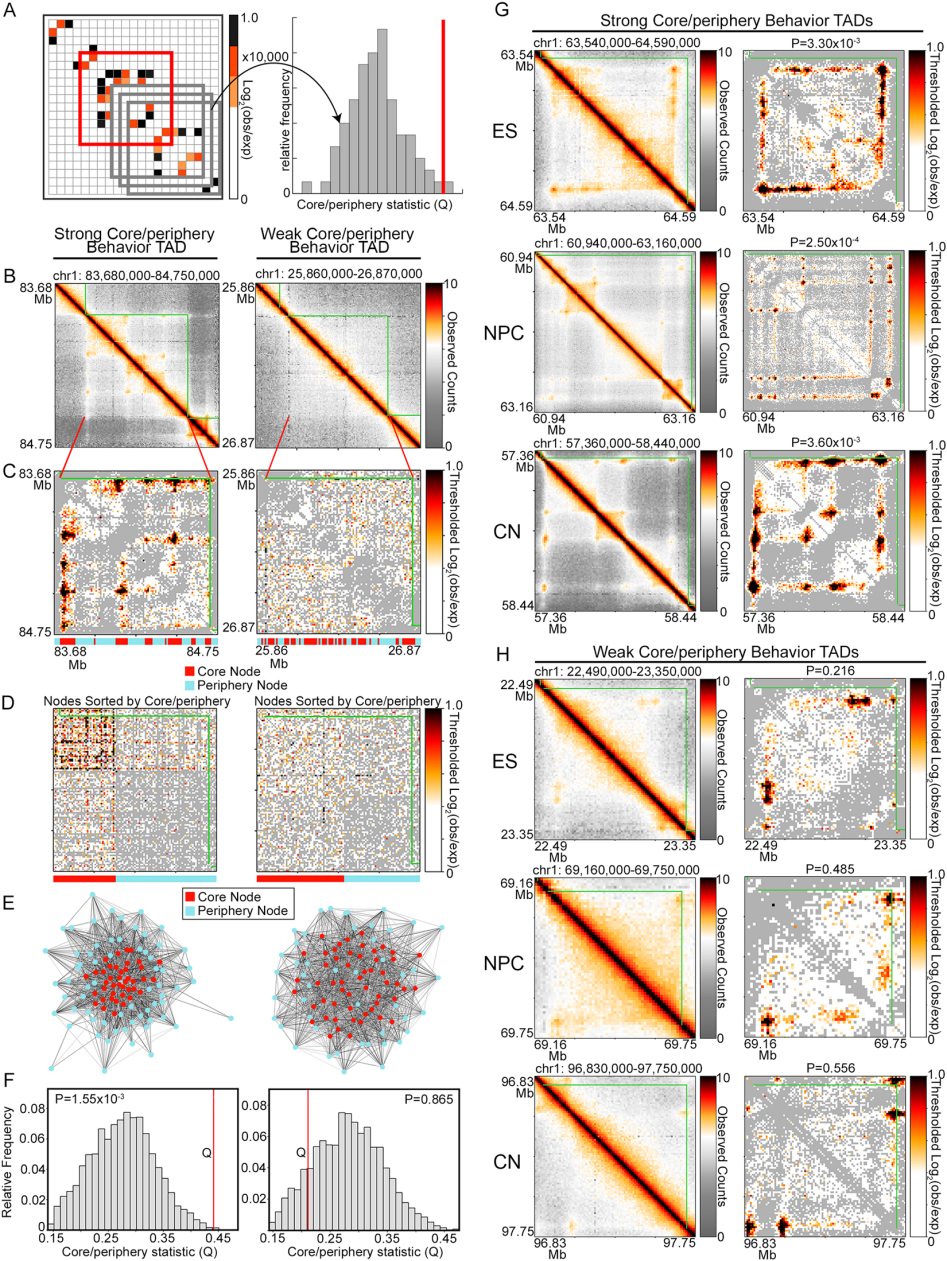
A subset of genome-wide TADs exhibit significant core-periphery network structure. (**A**) A TAD’s core-periphery test statistic Q (red) is compared to a null distribution of Q values from 10,000 randomly placed windows of equal size on the same chromosome (gray). The one-tailed empirical p-value is calculated as the percentage of random Q values greater than or equal to the real TAD Q value. (**B**) Observed heatmaps of a strong (left column) and weak (right column) core-periphery TAD in embryonic stem cells. (**C**) Observed/Expected heatmaps for TADs in panel (B) with negative interaction scores thresholded to 0. Thresholded values are shown in gray. Locations of core and periphery nodes are shown below each heatmap. (**D**) Observed/Expected heatmaps from panel (C) sorted to align all core-core edges at the top left and all periphery-periphery edges at the bottom right. Locations of core and periphery nodes are shown below each heatmap. (**E**) Kamada-Kawai force-directed graph visualizations of the TADs in panel (C). Nodes are colored according to their core-periphery classification. Red, core. Blue, periphery. (**F**) Null distribution and test statistic for each respective TAD in panel (C). (**G**-**H**) Observed heatmaps of TADs exhibiting (**G**) strong, significant and (**H**) weak core-periphery behavior in each cell type. Empirical p-values are listed on each Observed/Expected heatmap.

We next set out to identify  chromatin domains genome-wide that exhibit the highest core-periphery network structure in ES cells, NPCs, and cortical neurons. For each individual TAD network in each independent cell type, we compared its core-periphery test statistic, Q, to a null distribution of Q values computed on 10,000 randomly placed, size-matched genomic intervals (Fig. 2A, Supplementary Methods). We rejected the null hypothesis that the specific TAD does not exhibit core-periphery structure in cases where the multiple-testing corrected empirical p-value was less than a rigorous threshold of alpha <= 0.012 (Fig. 2A, Supplementary Methods). We identified a subset of TADs with the strongest possible core-periphery structure in mouse ES cells, NPCs, and cortical neurons (Supplementary Fig. 4) and compared their structural features to TADs that were not significantly core-periphery. We noticed that strong core-periphery TADs contain nodes that form multiple looping events with other highly looping nodes (Fig. 2B–F left column, Fig. 2G). By contrast, TADs devoid of core-periphery network structure are either depleted of looping interactions or contain single anchor-to-anchor looping interactions that do not involve multiple nodes (Fig. 2B–F right column, Fig. 2H). Importantly, we observed that core nodes directly correspond to genomic fragments engaged in multiple, highly connected looping interactions. These results reveal that a gradient of core-periphery network architectures can be found across TADs genome-wide, with complex core-periphery TADs containing complex hubs of hyper-connected genomic anchors engaged in multiple long-range loops.

We next sought to understand the differences between core and periphery nodes by integrating epigenetic annotations on the linear genetic sequence with all nodes in TADs with significantly strong core-periphery network architecture. We analyzed CTCF and H3K27ac ChIP-seq data from Cavalli and colleagues in the same mouse ES cells, NPCs, and cortical neurons used to generate the Hi-C data (Supplementary Table 8). Specifically, we stratified (1) CTCF occupancy into seven cell type-specific classes, including: ES-only, NPC-only, CN-only, ES + NPC, ES + CN, NPC + CN, and constitutively bound sites, (2) six classes of cell type-specific putative enhancers exhibiting positive H3K27ac signal and distal from transcription start sites, including: ES-only, NPC-only, CN-only, ES + NPC, ES + CN, and NPC + CN putative enhancers, and (3) eight classes of genes exhibiting positive H3K27ac signal at their transcription start sites, including: ES-only, NPC-only, CN-only, ES + NPC, ES + CN, NPC + CN, constitutively active, and constitutively inactive (Supplementary Tables 9–29, Supplementary Methods). As previously reported^35^, the large majority of CTCF bound sites (n = 44,830) are ES cell-specific, with neural differentiation involving a continuous trimming back of CTCF occupancy (Supplementary Fig. 5A–D). We observed a notable group of constitutively occupied CTCF sites across all three lineages (n = 8,853) and negligible CN-specific CTCF sites. By contrast, there were notable groups of ES cell-specific (n = 13,634), NPC-specific (n = 1,088), and CN-specific (n = 6,343) putative enhancers marked by strong H3K27ac+ signal distal from transcription start sites (Supplementary Figs 5E–H, 6). We also stratified groups of cell type-specific, inactive, and constitutively active genes by H3K27ac+ signal at transcription start sites (Supplementary Figs 5I–L, 6). These results confirm the known groups of cell type-specific genes and enhancers and that CTCF occupancy largely falls into a class of ES cell-specific or constitutive occupancy across cell types.

**Figure 3:**
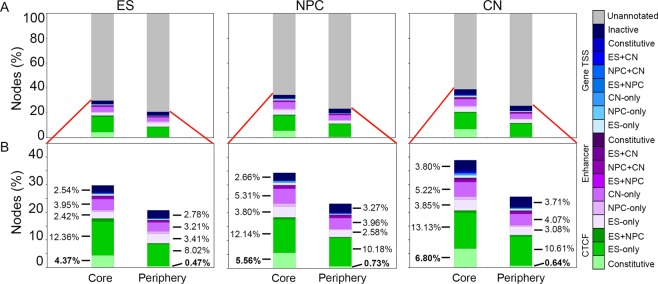
Distribution of 1-D Epigenome annotations with respect to core and periphery nodes. (**A**) Core and periphery nodes in significantly core-periphery TADs in ES cells, NPCs, and CNs were intersected with genome-wide cell type-specific CTCF occupied sites, putative enhancers, and genes. (**B**) Zoom-in of the percent of core and periphery nodes intersecting annotations in TADs with significant core-periphery network structure.

**Figure 4:**
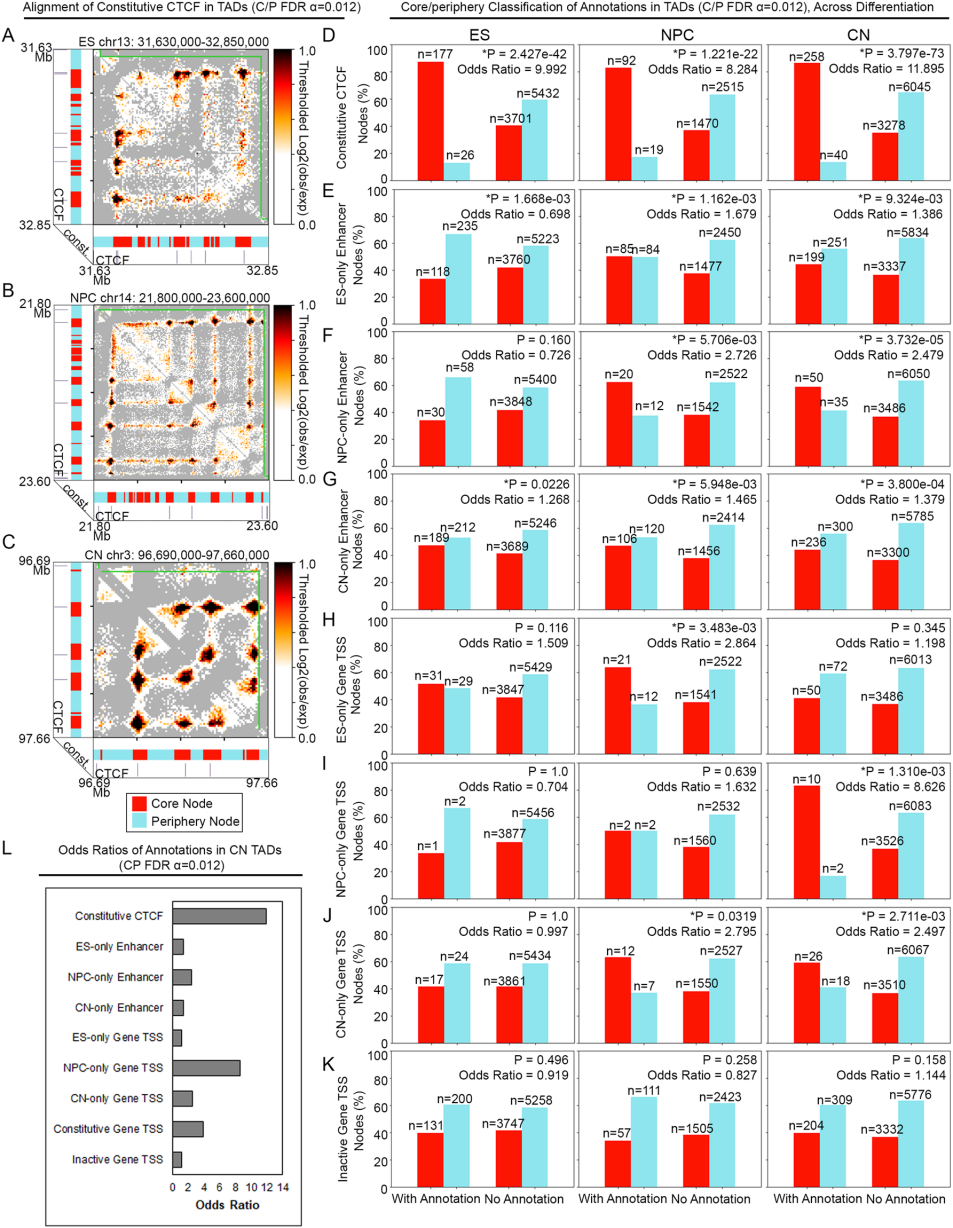
Constitutive and cell-specific CTCF occupied sites are strongly enriched at core vs. periphery nodes across cell types. (**A**–**C**) Observed/Expected heatmap examples in (**A**) ES cell (**B**) NPC and (**C**) CN TADs with significant core-periphery architecture illustrating co-localization of constitutive CTCF occupancy with core nodes and not with periphery nodes. (**D**–**K**) Bar plots comparing proportions of core and periphery nodes that intersect the annotation of interest vs. do not intersect the annotation of interest. Odds ratios (OR) are reported for all plots. (**D**) Constitutive CTCF, (**E**) ES-only enhancers, (**F**) NPC-only enhancers, (**G**) CN-only enhancers, (**H**) ES-only genes, (**I**) NPC-only genes, (**J**) CN-only genes, (**K**) Inactive genes. (**L**) Summary of Odds ratios for 1-D Epigenome annotation enrichment in core vs. periphery nodes for all TADs with significant core-periphery structure in cortical neurons.

We next canvased the set of all possible cell type-specific annotations for their co-localization at core versus periphery nodes in TADs with significant core-periphery network structure. We found that core nodes were markedly more co-localized with CTCF occupied sites than periphery nodes (Fig. 3). Consistent with this observation, constitutively bound CTCF sites were strongly enriched for core nodes and depleted for periphery nodes across core-periphery TADs from all three cell types (Fig. 4A–D, Supplementary Fig. 7). Nearly all cell type-specific enhancer and gene classes were evenly distributed between core and periphery nodes with modest Odds Ratios (Fig. 4E–L, Supplementary Figs 8–9, Supplementary Tables 30–32). The one exception was constitutively expressed genes significantly enriched in core nodes versus periphery nodes in cortical neuron core-periphery TADs at our stringent p-value cutoff of P<0.0005 (Odds Ratio = 3.977) (Fig. 4L, Supplementary Fig. 9). Taken together, these results reveal that hyper-connected core genome folding nodes are occupied by CTCF and that cell type-specific enhancers and genes show no clear bias to core vs. periphery nodes in TADs with core-periphery network structure.

**Figure 5:**
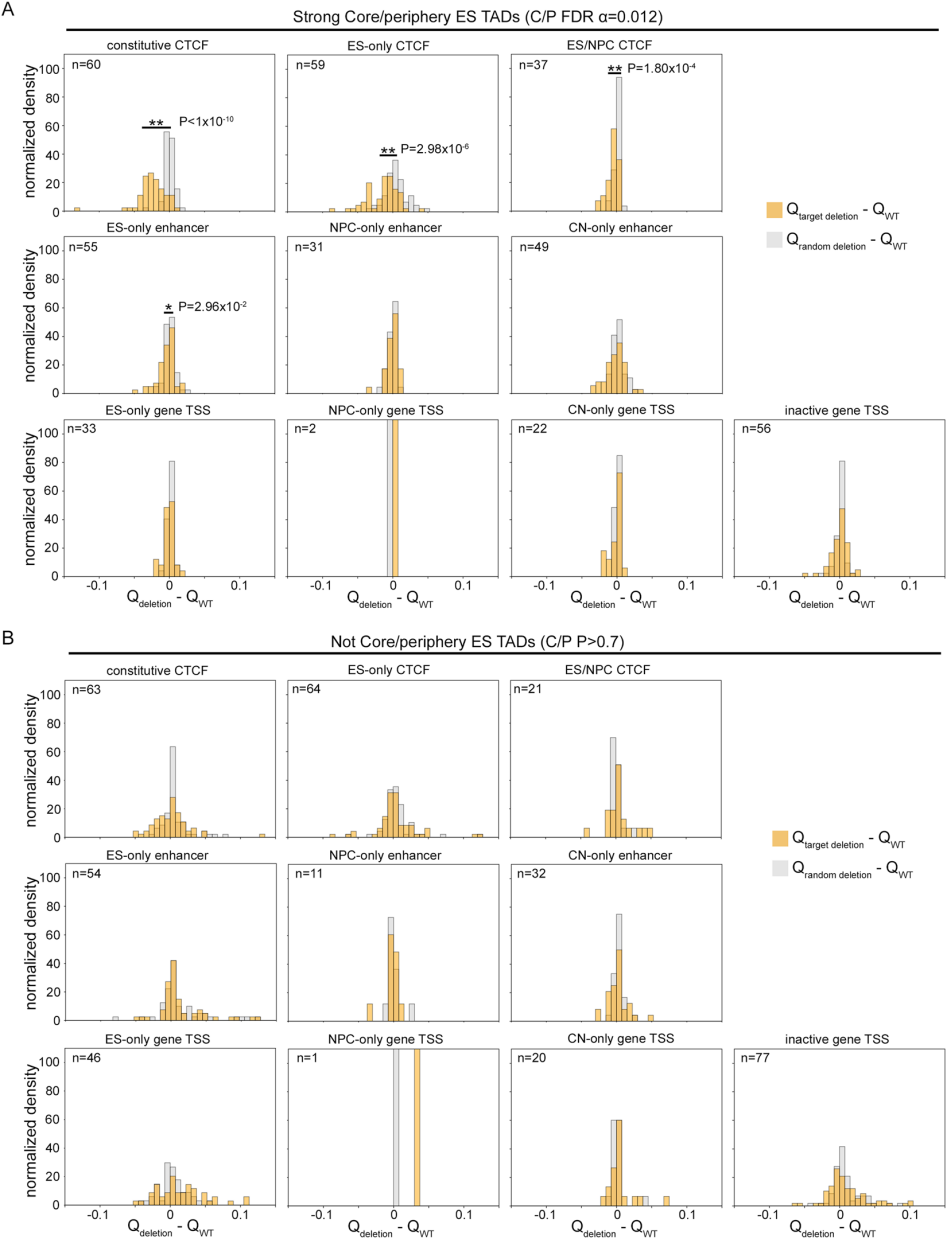
*In silico* mutation of CTCF occupied nodes results in significant ablation of core-periphery network structure. (**A**) Difference in Q values before and after node deletion in significant core-periphery TADs for three classes of CTCF (top), three classes of enhancers (middle), and four classes of gene TSS (bottom). We deleted nodes that co-localize with target 1-D Epigenome marks (orange) or the same number of random nodes (grey) for each TAD in ES cells. A single asterisk indicates a marginally significant difference in distribution means (two-tailed Mann-Whitney U test, P < 0.05), and a double asterisk indicates a highly significant difference in distribution means (two-tailed Mann-Whitney test U test, P < 0.005). (**B**) The same analysis was performed for TADs in ES cells that do not exhibit core-periphery network structure (Supplementary Methods). No significant difference was found for any target annotation (two-tailed Mann-Whitney U test).

**Figure 6:**
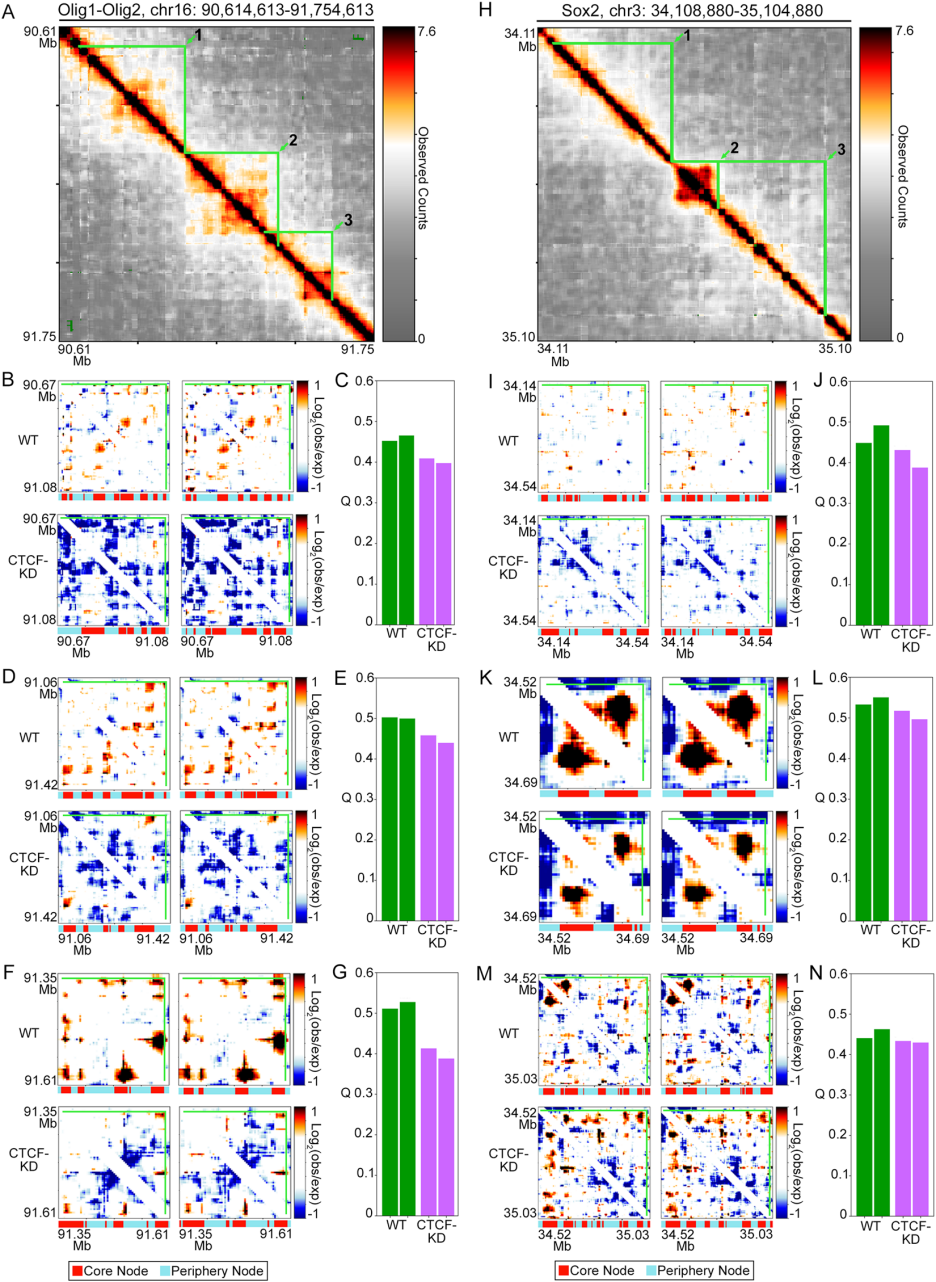
Knockdown of CTCF levels in ES cells results in decreased core-periphery network structure in subTADs around pluripotency genes. (**A**) Observed heatmap of Chromosome-Conformation-Capture-Carbon-Copy data around the Olig1 and Olig2 genes in ES cells. The three Olig1-Olig2 subTADs used in subsequent core-periphery analysis are outlined in green and labeled 1 (**B**,**C**), 2 (**D**,**E**), and 3(**F**,**G**). (**B**,**D**,**F**) Observed/Expected heatmaps of Olig1-Olig2 (**B**) subTAD 1, (**D**) subTAD 2, and (**F**) subTAD3 in two wild type ES cell replicates (to row) and two CTCF knock down ES cell replicates (bottom). (**C**,**E**,**G**) Core-periphery test statistic Q for (**C**) subTAD 1, (**E**) subTAD 2, and (**G**) subTAD3. (**H**) Observed heatmap of Chromosome-Conformation-Capture-Carbon-Copy data around the Sox2 gene in ES cells. The three Sox2 subTADs used in subsequent core-periphery analysis are outlined in green and labeled 1 (**I**,**J**), 2 (**K**,**L**), and 3(**M**-**N**). (**I**,**K**,**M**) Observed/Expected heatmaps of Sox2 (**I**) subTAD 1, (**K**) subTAD 2, and (**M**) subTAD3 in two wild type ES cell replicates (to row) and two CTCF knock down ES cell replicates (bottom). (**J**,**L**,**N**) Core-periphery test statistic Q for (**J**) subTAD 1, (**L**) subTAD 2, and (**N**) subTAD3. Core and periphery nodes are labeled under each heatmap.

To determine if looping core-periphery structure could be disrupted, we performed both *in silico* mutagenesis and *in vitro* experimental knock down experiments. We first conducted *in silico* mutagenesis experiments by computing the core-periphery test statistic Q after either deleting nodes containing a particular 1-D Epigenomic annotation or deleting nodes at random (Fig. 5, Supplementary Fig. 10). We found that *in silico* mutation of CTCF binding sites significantly disrupted core-periphery structure in both ES cells (Fig. 5A) and CNs (Supplementary Fig. 10A). This effect was specific to significantly core-periphery TADs and was not made manifest in the same analysis on TADs that did not exhibit notable core-periphery structure (Fig. 5B, Supplementary Fig. 10B). Moreover, *in silico* mutation of cell type-specific enhancers or genes had a minimal effect on core-periphery structure. We also knocked down CTCF to 20% of its wild type levels in mouse ES cells using lentiviral shRNA (Supplementary Fig. 11, Supplementary Table 33). We compared genome folding in wild type and CTCF knock-down ES cells by using Chromosome-Conformation-Capture-Carbon-Copy (5C) as previously described^10,35–39^. Although CTCF levels were not completely ablated, we found that network core-periphery structure was slightly reduced in some but not all chromatin domains (Fig. 6). Together, these results demonstrate that core-periphery network structure in a subset of TADs can be altered upon disruption of CTCF protein levels or mutagenesis of CTCF’s occupied sites in the genome.

**Figure 7:**
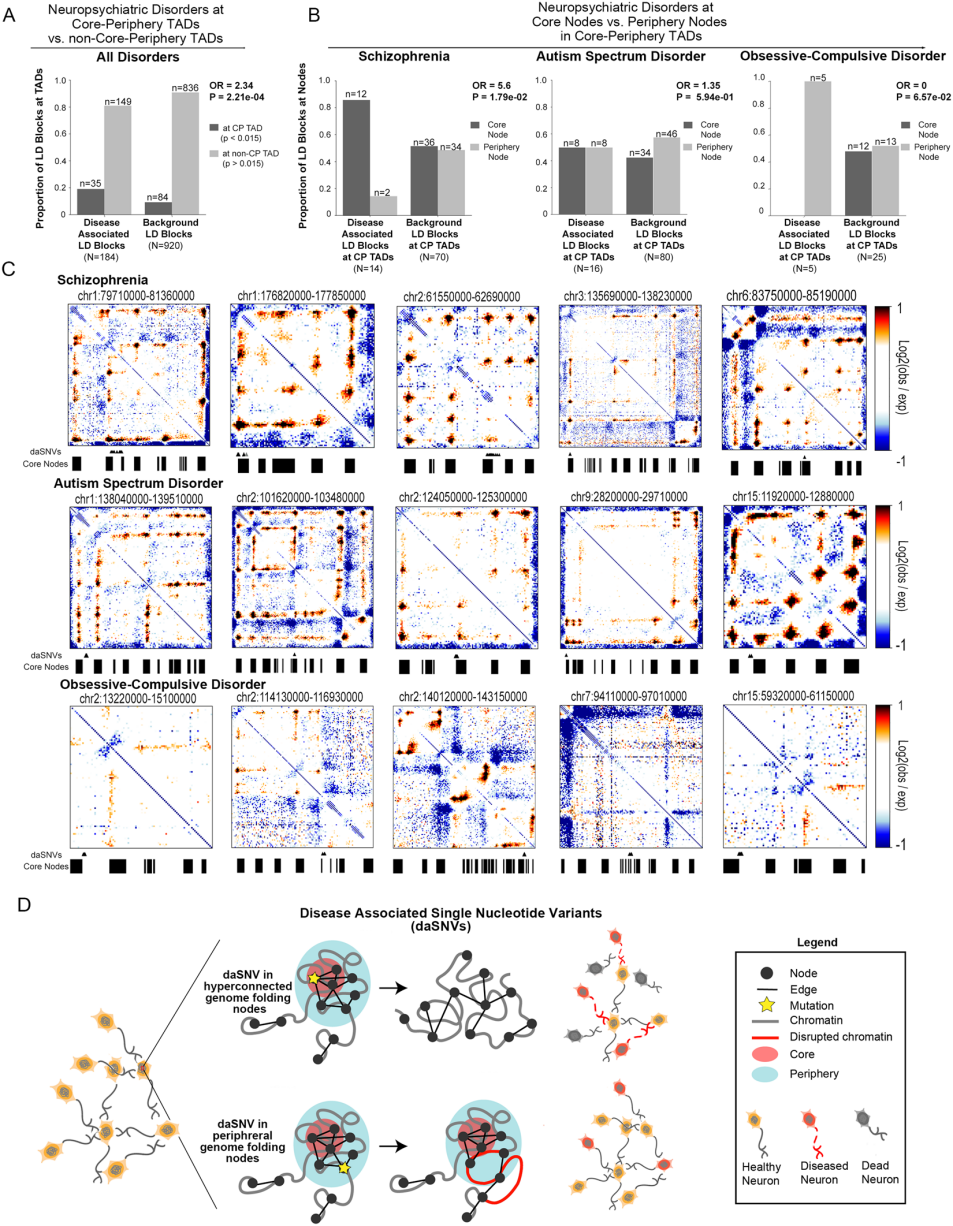
TADs in cortical neurons with strong core-periphery mesoscale structure are enriched for neuropsychiatric disease-associated single nucleotide variants. (**A**) Odds ratios representing the enrichment of schizophrenia-, OCD-, and ASD-associated common single nucleotide variants (SNVs) in TADs with strong and weak core-periphery structure compared to linkage disequilibrium size- and minor allele frequency-matched background SNVs (N = 100 sets of background SNVs). Pvalues, Fisher’s exact test. (**B**) Odds ratios representing the enrichment of schizophrenia-, OCD-, and ASD-associated common SNVs at core versus periphery nodes in TADs with strong core-periphery structure compared to linkage disequilibrium size- and minor allele frequency-matched background SNVs (N = 100 sets of background SNVs). Pvalues, Fisher’s exact test. (**C**) Observed/Expected heatmaps of neuropsychiatric daSNVs at core and periphery nodes in TADs with strong core-periphery structure. daSNV LD blocks are plotted below the heatmap (Supplementary Methods). (**D**) Schematic working model of the core-periphery network structure of the 3-D genome and our hypothesized model of the topological and functional consequences of daSNVs at core versus periphery nodes.

Finally, we assessed the possible link between the 3-D genome’s core-periphery network structure and neuropsychiatric disease-associated single nucleotide variants (daSNVs). We first observed that TADs in CNs with significant core-periphery structure are significantly more conserved across placental mammals than domains devoid of such structure (Supplementary Fig. 12A). Core nodes in highly core-periphery TADs have significantly higher conservation and gene density compared to periphery nodes in core-periphery TADs (Supplementary Fig. 12B,C). Importantly, we observed that schizophrenia, ASD,  and OCD daSNVs in non-coding genomic regions were significantly more likely to co-localize with CN TADs with significant core-periphery structure than to CN TADs devoid of such structure (Fig. 7A, Supplementary Methods). We then compared the distribution of neuropsychiatric daSNVs across core and periphery genome folding nodes (Fig. 7B,C). Importantly, we found that the linkage disequilibrium (LD) blocks encompassing schizophrenia daSNVs were significantly enriched at core nodes, wheras OCD daSNVs were enriched at periphery nodes, and ASD daSNVs were equally distributed across core or periphery nodes (Fig. 7B,C). Size-matched, background SNV LD blocks exhibited a relatively even distribution across core and periphery nodes (Fig. 7B, Supplementary Methods). We hypothesize that daSNVs disrupting highly interconnected core nodes would result in severe topological and functional disruption leading to severe pathological phenotypes (Fig. 7D). We also posit that daSNVs in the periphery of genome folding networks might preserve global genome folding but result in slight local topological and functional disruptions that contribute in aggregate to human disease (Fig. 7D).

## Discussion

Complex systems from a wide range of orthogonal disciplines can be represented as networks. Networks are defined mathematically as a collection of nodes and the pattern of edges between them. Because data from genome-wide proximity ligation studies are traditionally represented in the form of a square, symmetric array, we reasoned that computational tools and mathematical models from network science might have utility in capturing mesoscale structure not visible to the human eye in maps of higher-order chromosome architecture. Previously, by applying graph theory methods to Hi-C data, we found that the topology of the mammalian genome is organized into nested community structures that correspond to TADs and subTADs^31^. Here, we again demonstrate that graph theory tools have utility in uncovering an additional layer of mesoscale genome folding. We find that looping interactions in a subset of TADs and subTADs can fold into core-periphery network structures. Prior to this study it was unknown whether genome folding at the highest resolution within large scale TADs had a specific mesoscale network structure. Although network algorithms to query mesoscale structure have existed for some time, progress was limited by the paucity of high-resolution Hi-C data required to detect looping interactions genome-wide. Our finding of core-periphery network structure within a subset of chromatin domains was made possible by the very recent publication of the highest read depth Hi-C libraries to date across mouse ES cells, NPCs, and CNs^34^.

Together, our data suggest that understanding the mesoscale network structures of 3-D genome folding in a comprehensive, quantitative manner could shed new light into mechanisms governing how gene expression patterns are altered over long genomic distances by common genetic variation. First, we find that hyper-connected core nodes are highly enriched for the architectural protein CTCF and its knockdown leads to topological disruption of genome folding core-periphery networks. Second, in cortical neurons, we observed that neuropsychiatric daSNVs are co-localized with TADs exhibiting significant core-periphery network structure. Our working hypothesis is that daSNVs enriched at core looping nodes might lead to severe or early onset forms of human disease due to global disruption of large-scale chromatin architecture and gene expression (Fig. 7D). It is tempting to speculate that mutations in the periphery of genome folding networks might preserve global genome folding networks, but result in local topological and functional disruptions contributing slightly to human disease. In light of the growing knowledge that many complex traits and diseases are thought to be driven by a large number of common variants with small effect sizes^40–43^, an exciting area of future inquiry will be to understand the localization and functional importance of human genetic variation contributing to disease and cell type-specific gene expression signatures compared to core and periphery nodes in genome folding networks.

Core-periphery structure is abundant in a wide range of complex networks across disciplines, and has critical implications for system function and dynamics. Intuitively, the architecture itself allows for the top-down influence, or even control of, core regions on a receptive periphery. In social systems, core-periphery architecture is thought to support an optimal trade-off in the capacity for social groups to both broadcast (to the periphery) and receive (from the periphery) information critical to the group’s survival and success^44^. In information processing systems such as the brain, core-periphery structure is thought to support the ability of the core to integrate the multimodal information obtained from the periphery. In transportation systems, often dual or multiple cores are identified, possibly driven by constraints of geography and the evolution of the system (and the culture it supports) over time^30^.

Core-periphery structure also impacts the manner in which the system evolves over time and responds to exogenous perturbations^45^. In studies of population dynamics, cores are associated with relatively stable population dynamics, while increasing variance and importance of density-independent processes operate at the periphery^46^. Similar inferences have been drawn in the context of human brain dynamics, where a core of brain regions associated with task performance displays more stable dynamics than a periphery of brain regions associated with supportive roles in cognition^47^. Studies of population-level behavior in humans have also provided initial evidence that resources may be over-utilized in the network core, and under-utilized in the network periphery^48^. Finally, the architecture has important implications for robustness to failures, spreading dynamics, or collective behaviors across systems spanning biological, social, and technological domains^49^. Exciting areas of future inquiry will involve the causal studies necessary to determine how the functional roles for core-periphery structure in complex systems may be made manifest in TADs with core-periphery genome folding structure and human gene regulation.

Methods from graph theory and network science have recently been applied to find biologically meaningful patterns in chromatin folding data^31,32,50–56^. Multi-dimensional scaling has been used to convert 2-D proximity ligation matrices into 3-D models of the physical folding of chromosomes^50^. Ruan and colleagues assembled a binary network of chromatin interactions between RNA polymerase II bound genomic regions from ChIA-PET data^53^. They reported that RNA II-mediated connections in the human genome resemble a modular, scale-free network in which most nodes are weakly connected and then a small number of nodes have a high number of interactions. The authors hypothesized that chromatin networks may have evolved scale-free properties to better tolerate random perturbations^57^. Babaei and colleagues recently pursued the possibility of predicting co-expressed gene pairs by network metrics computed from mouse cortex Hi-C data such as the Jaccard index, the betweenness centrality, and the clustering coefficient^55^. Similarly, independent studies have explored genome folding patterns at master replication origins, histone modifications and cohesin binding sites using network metrics such as centrality, clustering coefficient, and assortativity^51,52,56^. Finally, we and others have also recently demonstrated that larger TADs and smaller subTADS can be parsed across length scales via the fine-tuning of the structural resolution parameter in a Louvain-like locally greedy algorithm to maximize a modularity quality function^31,32^. Although the concept of analyzing 3-D genome folding as a network has been pursued previously^31,32,50–56^, here we take an entirely new angle, which is to discover mesoscale core-periphery network structure in a subset of mammalian TADs. The ability to accurately quantify core-periphery network structure in Hi-C genome folding maps provides future opportunities to shed new light on how genome sequence influences 3-D structure to govern function across development and during the onset and progression of disease.

## Supplementary information

Supplemental Figures, Tables, Methods

Supplementary Table 2

Supplementary Table 3

Supplementary Table 4

Supplementary Table 5

Supplementary Table 6

Supplementary Table 7

Supplementary Table 9

Supplementary Table 10

Supplementary Table 11

Supplementary Table 12

Supplementary Table 13

Supplementary Table 14

Supplementary Table 15

Supplementary Table 16

Supplementary Table 17

Supplementary Table 18

Supplementary Table 19

Supplementary Table 20

Supplementary Table 21

Supplementary Table 22

Supplementary Table 23

Supplementary Table 24

Supplementary Table 25

Supplementary Table 26

Supplementary Table 27

Supplementary Table 28

Supplementary Table 29

## Data Availability

Data in this manuscript were downloaded from GEO as detailed in the Supplementary Methods. We will freely share code upon request.
