# Supplementary material for: A subset of topologically associating domains fold into mesoscale core-periphery networks
Source: Sci Rep. 2019 Jul 2;9:9526. doi: 10.1038/s41598-019-45457-9 (PMC6606598; doi:10.1038/s41598-019-45457-9)
Supplement: Supplementary file 1 — Supplemental Figures, Tables, Methods [file 41598_2019_45457_MOESM1_ESM.docx]

**A subset of topologically associating domains fold into mesoscale core-periphery networks**

**Harvey Huang^1*^, Sunny Chen^1*^, Katelyn R. Titus^1*^, Daniel Emerson^1^,**

**Danielle S. Bassett^1,4,5,6^, Jennifer E. Phillips-Cremins^1,2,3**^**

**^1^Department of Bioengineering, School of Engineering and Applied Sciences, University of Pennsylvania, Philadelphia, PA 19104**

**^2^Epigenetics Institute, Perelman School of Medicine, University of Pennsylvania, Philadelphia, PA 19104**

**^3-D^epartment of Genetics, University of Pennsylvania, Philadelphia, PA 19104**

**^4^Department of Electrical & Systems Engineering, School of Engineering and Applied Sciences, University of Pennsylvania, Philadelphia, PA 19104**

**^5^ Department of Neurology, Perelman School of Medicine, University of Pennsylvania, Philadelphia, PA 19104**

**^6^Department of Physics & Astronomy, College of Arts and Sciences, University of Pennsylvania, Philadelphia, PA 19104**

***Authors contributed equally to this work
**Corresponding author: jcremins@seas.upenn.edu**

**SUPPLEMENTAL FIGURES**

| 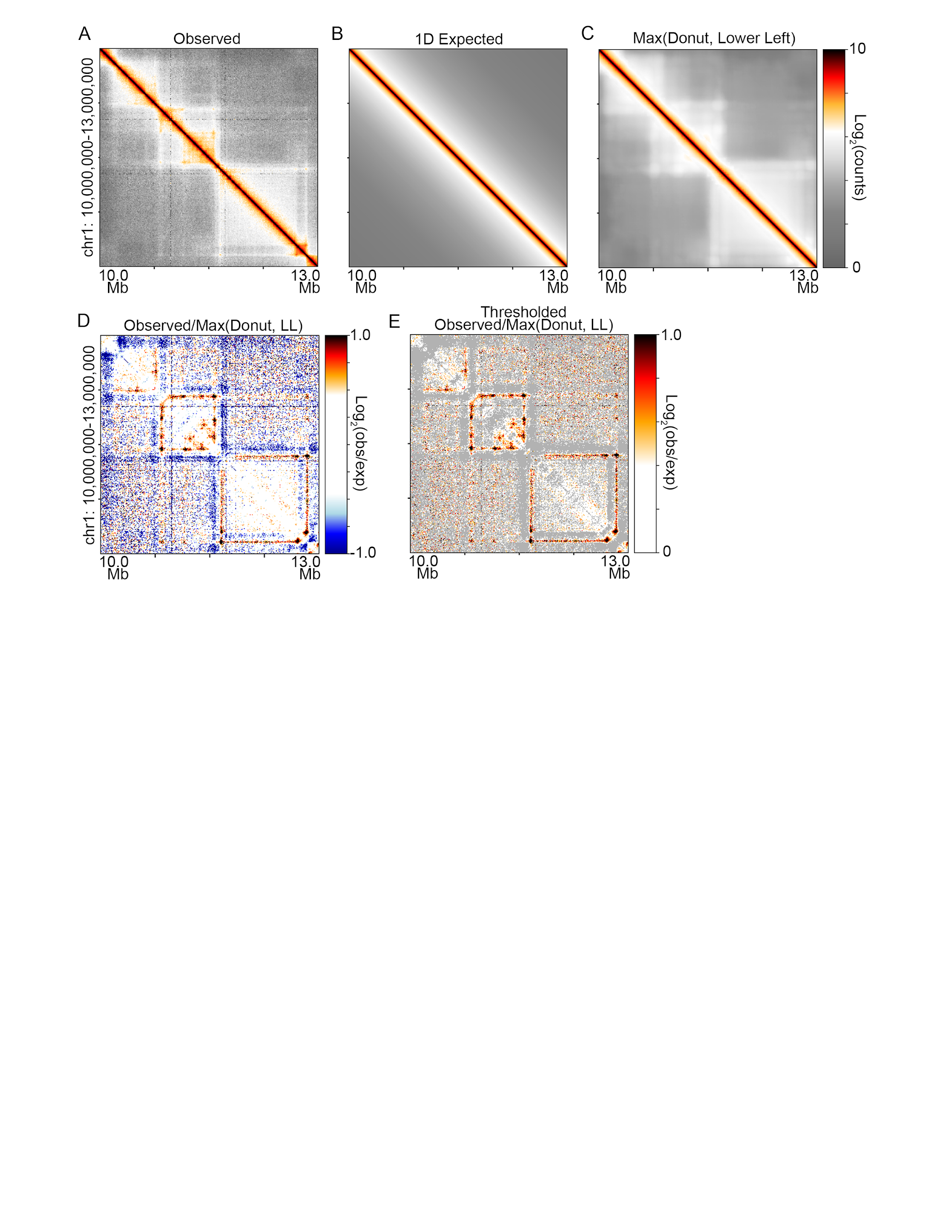 |
| --- |
| **Supplementary Figure 1. Hi-C data processing steps from observed data to thresholded observed/max(donut, lower left).** (A) An example of an observed counts contact heatmap, 3 megabase in size. Counts are shown in log base 2. (B) The distance-dependent expected model of A, showing expected counts based on linear proximity of genomic bins . (C) The max(donut, lower left) expected model of A, which models expected genome architecture. Distance dependency and underlying TAD architecture are captured in the model. (D) Observed-over-expected contact heatmap of A, where the observed counts were taken over the max(donut, LL) expected counts. Long-range chromatin interactions can now be visualized. (E) Thresholded contact heatmap of D, where all counts less than or equal to 0 (shown in gray) were thresholded to 0. |

| 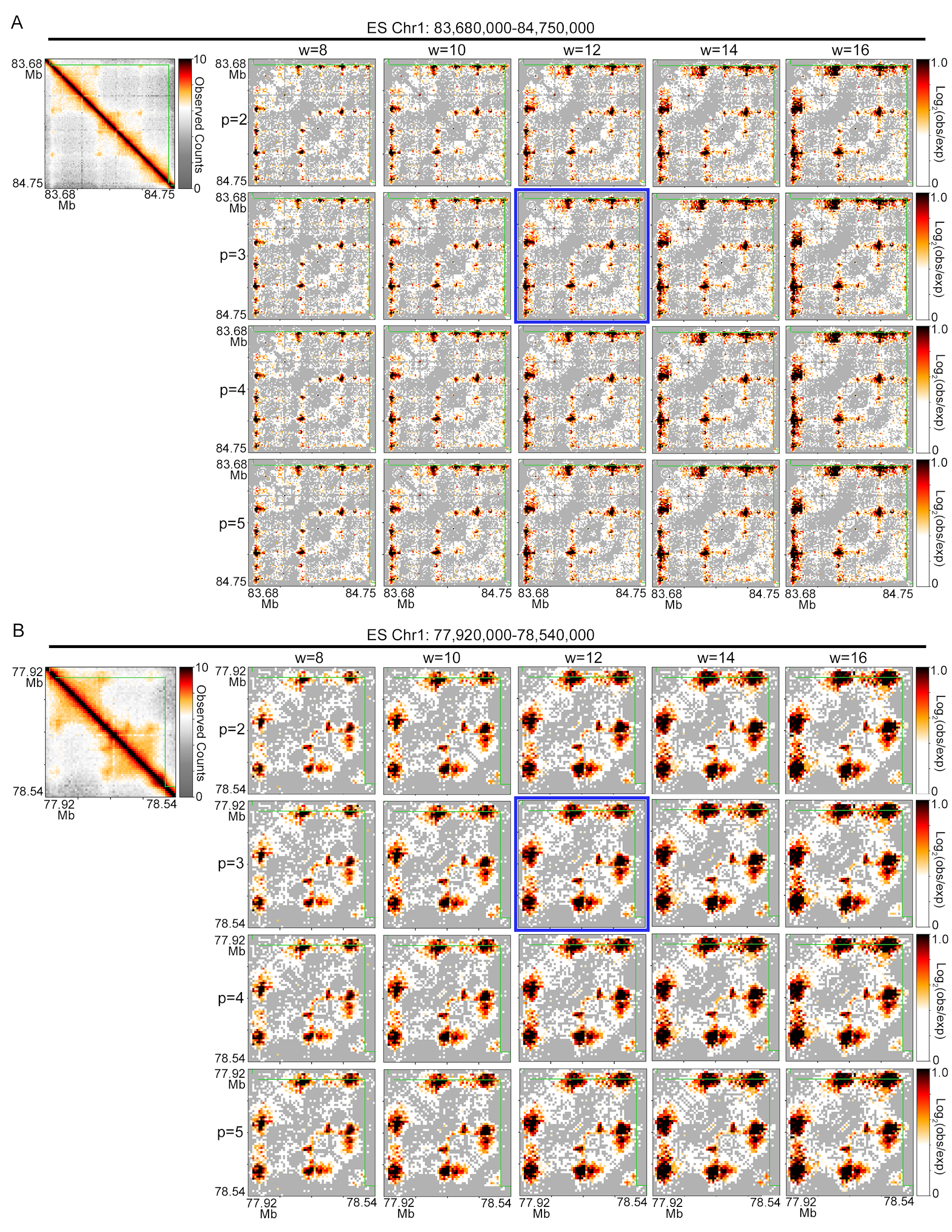 |
| --- |
| **Supplementary Figure 2. Values for the donut and lower left expected model p and w parameters were selected by means of visual inspection across a range of likely candidates.** (A) Observed heatmap of a large TAD (left), with corresponding observed-over- expected heatmaps of the same TAD at p=2,3,4,5 and w=8,10,12,14,16 combinations (right). The TAD boundaries are outlined in green. The heatmap using the selected combination of p=3, w=12 is outlined in blue. (B) Observed heatmap of a medium-sized TAD (left), with corresponding observed-over-expected heatmaps of the same TAD at p=2,3,4,5 and w=8,10,12,14,16 combinations (right). The TAD boundaries are outlined in green. The heatmap using the selected combination of p=3, w=12 is outlined in blue. |

| 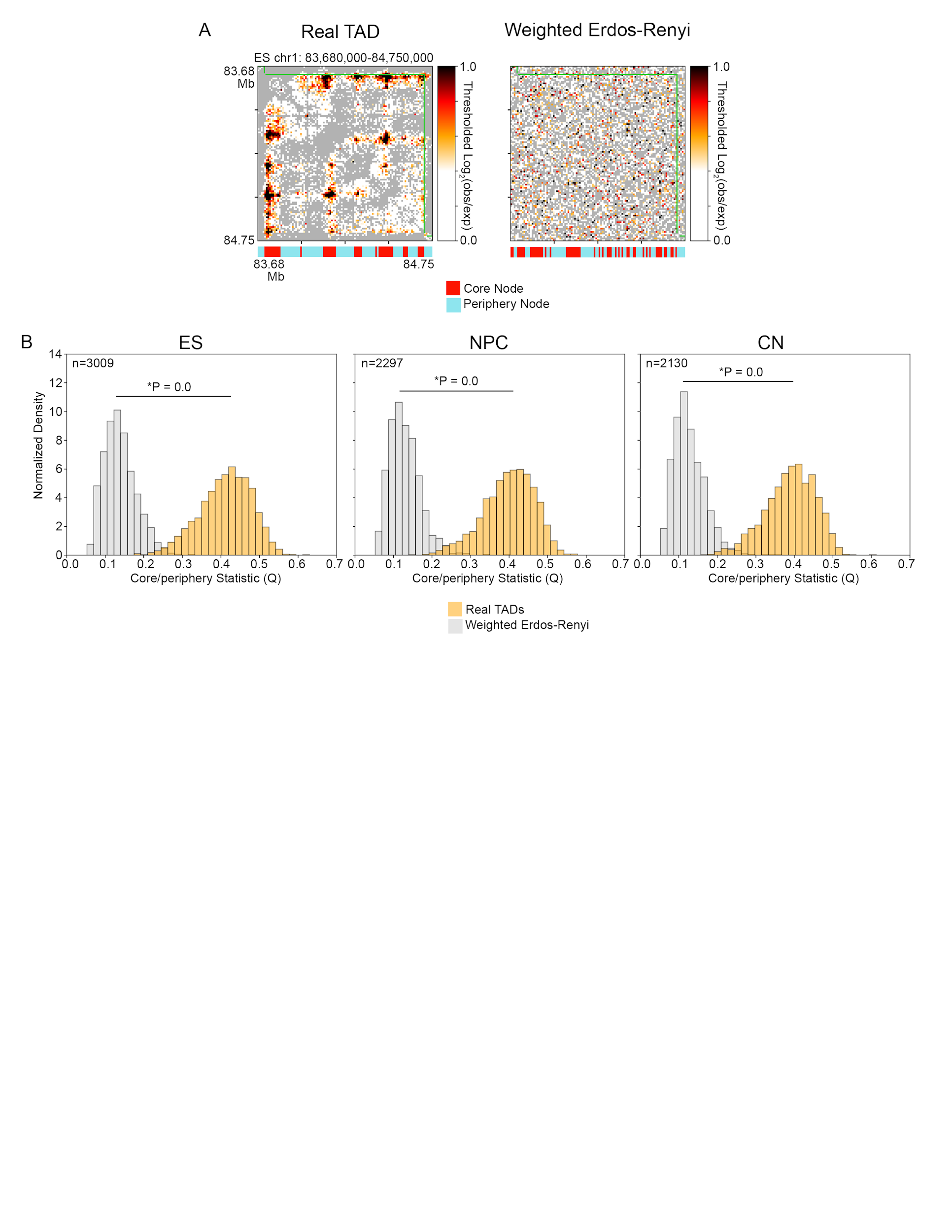 |
| --- |
| **Supplementary Figure 3. Long-range chromatin interactions within TADs show significantly stronger core-periphery structure than random networks.** (A) Comparison of a real TAD with weighted Erdős–Rényi  (ER) random networks of the same degree distribution. (B) Q distributions of real TADs versus weighted ER random networks for each cell type, where each random network is matched in degree distribution to a real TAD. Real TADs show much stronger core-periphery behavior than the random networks (ES K-S P=0.0, n=3009, NPC K-S P=0.0, n=2297, CN K-S P=0.0, n=2130). |

| 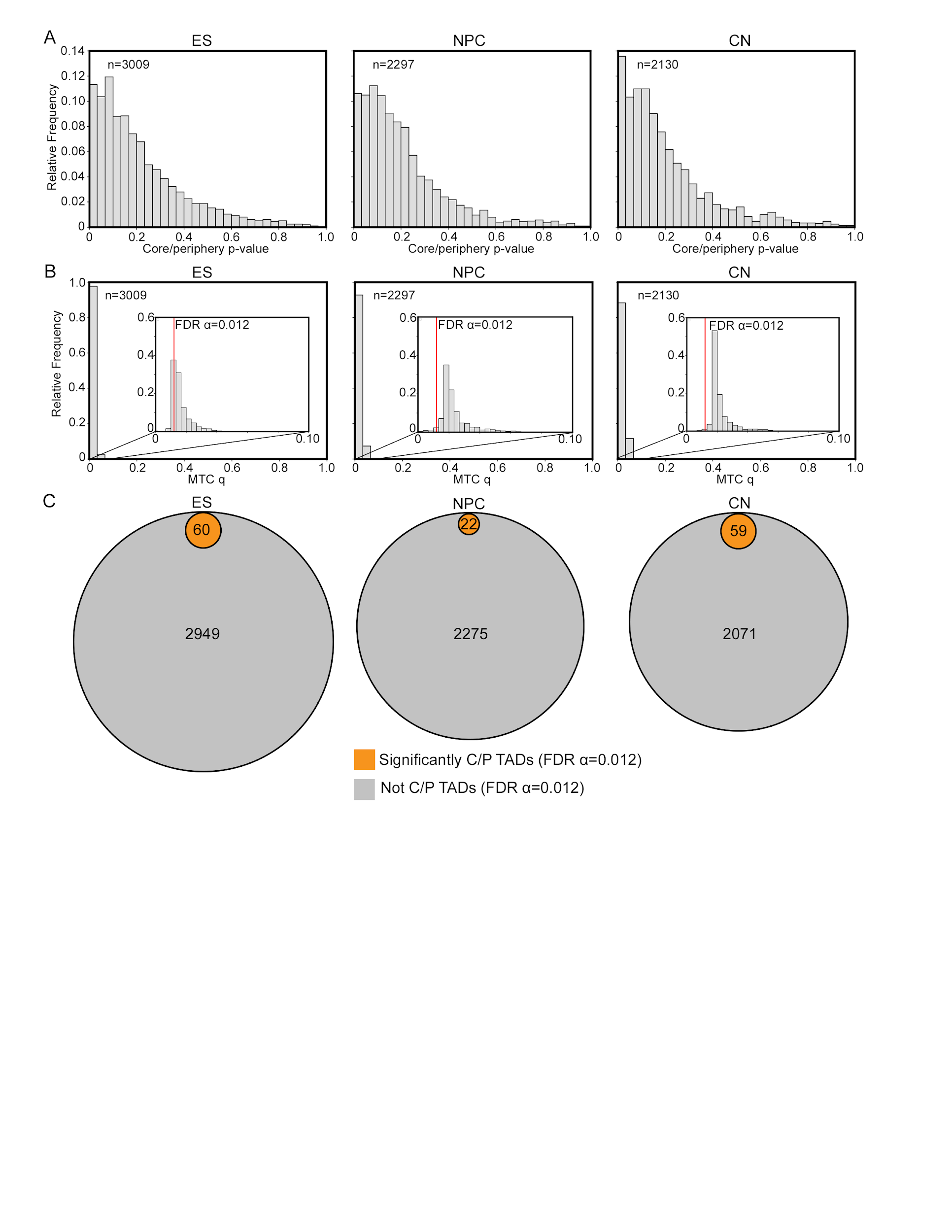 | |
| --- | --- |
| **Supplementary Figure 4. TADs exhibit gradually varying degrees of core-periphery behavior.** (A) Core-periphery p-value distributions of TADs in ES, NPC, and CN, before multiple testing correction. (B) Distribution of q after Storey/Tibshirani multiple testing correction. TADs that have a false disovery rate < 1.2% are selected as significantly core-periphery TADs. (C) The number of significantly core-periphery TADs (ES: n=60, NPC: n=22, CN: n=59) and non-significantly core-periphery TADs (ES: n=2949, NPC: n=2275, CN: n=2071). |  |

| 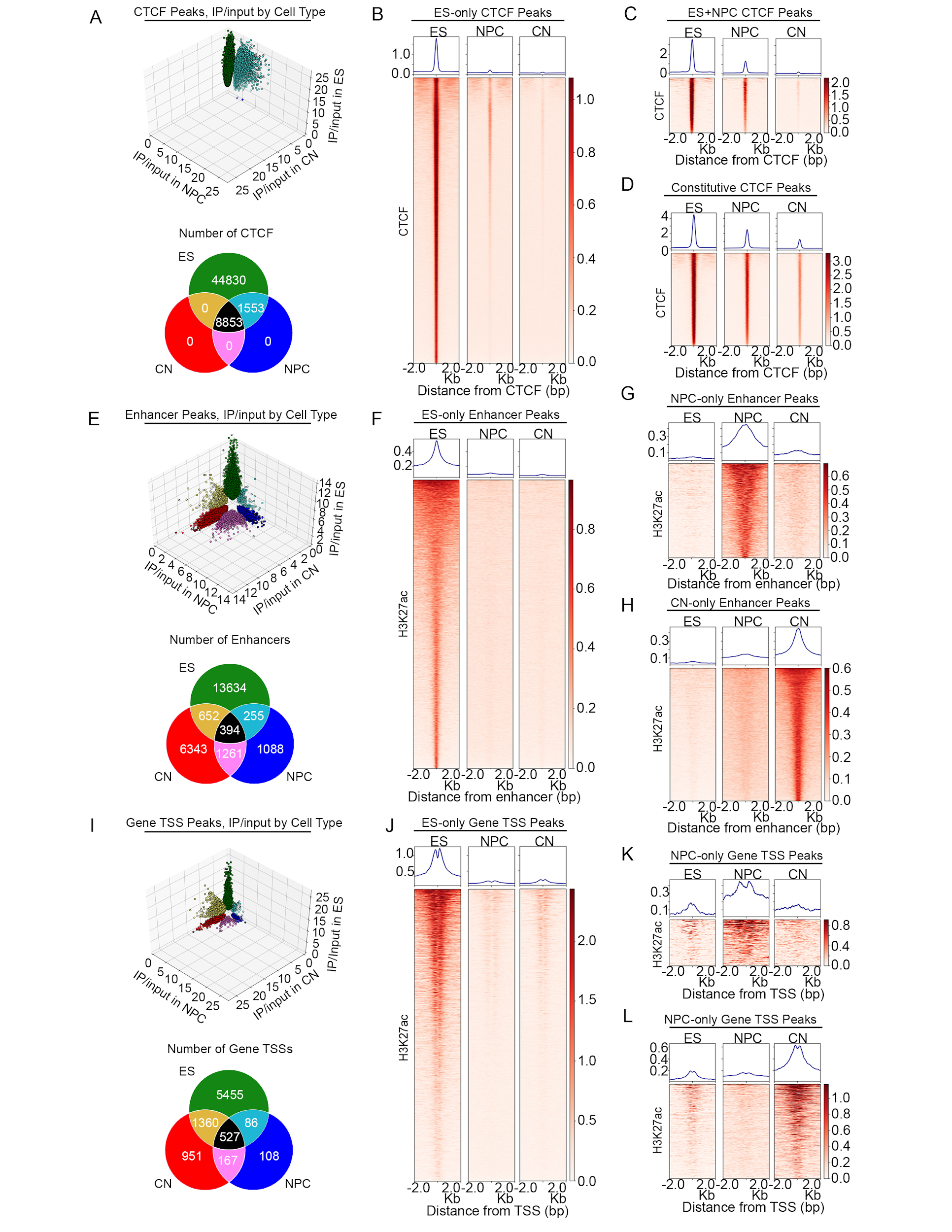 |
| --- |
| **Supplementary Figure 5. ChIP-seq peaks for CTCF, putative enhancer H3K27ac, and gene TSS H3K27ac are processed by signal thresholding for cell-type specific CTCF, enhancer, and gene annotations.** (A) 3D IP/input plot across ES, NPC, and CN for identified CTCF peaks. Number of cell-type specific CTCF annotations across cell type classes are plotted by Venn diagram. (B) ES-only CTCF ChIP-seq pileups and signal expression heatmaps across cell types. (C) NPC-only CTCF ChIP-seq pileups and signal expression heatmaps across cell types. (D) CN-only CTCF ChIP-seq pileups and signal expression heatmaps across cell types. (E) 3D IP/input plot across ES, NPC, and CN for enhancer H3K27ac peaks. Number of cell-type specific enhancer annotations across cell type classes are plotted by Venn diagram. (F) ES-only enhancer ChIP-seq pileups and signal expression heatmaps across cell types. (G) NPC-only enhancer ChIP-seq pileups and signal expression heatmaps across cell types. (H) CN-only enhancer ChIP-seq pileups and signal expression heatmaps across cell types. (I) 3D IP/input plot across ES, NPC, and CN for gene TSS H3K27ac peaks. Number of cell-type specific gene TSS annotations across cell type classes are plotted by Venn diagram. (J) ES-only gene ChIP-seq pileups and signal expression heatmaps across cell types. (K) NPC-only gene ChIP-seq pileups and signal expression heatmaps across cell types. (L) CN-only gene ChIP-seq pileups and signal expression heatmaps across cell types. |

| 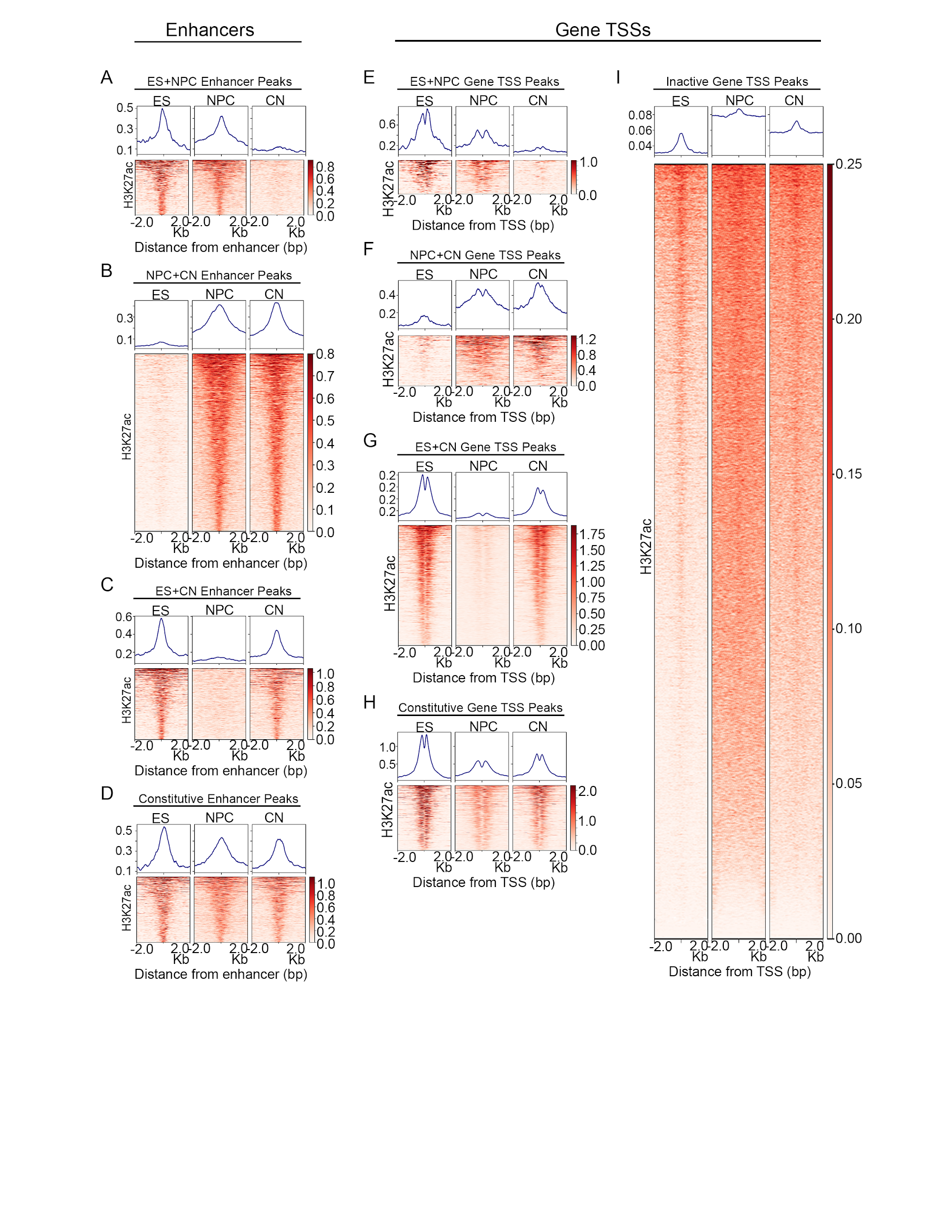 |
| --- |
| **Supplementary Figure 6. Annotation ChIP-seq signal pileups and heatmaps for enhancer and gene TSS two-way cell classifications, constitutive, and inactive classifications.** (A) ES+NPC enhancer ChIP-seq pileups and signal expression heatmaps across cell types. (B) NPC+CN enhancer ChIP-seq pileups and signal expression heatmaps across cell types. (C) ES+CN enhancer ChIP-seq pileups and signal expression heatmaps across cell types. (D) Constitutive enhancer ChIP-seq pileups and signal expression heatmaps across cell types. (E) ES+NPC gene TSS ChIP-seq pileups and signal expression heatmaps across cell types. (F) NPC+CN gene TSS ChIP-seq pileups and signal expression heatmaps across cell types. (G) ES+CN gene TSS ChIP-seq pileups and signal expression heatmaps across cell types. (H) Constitutive gene TSS ChIP-seq pileups and signal expression heatmaps across cell types. (I) Inactive gene TSS ChIP-seq pileups and signal expression heatmaps across cell types. |

| 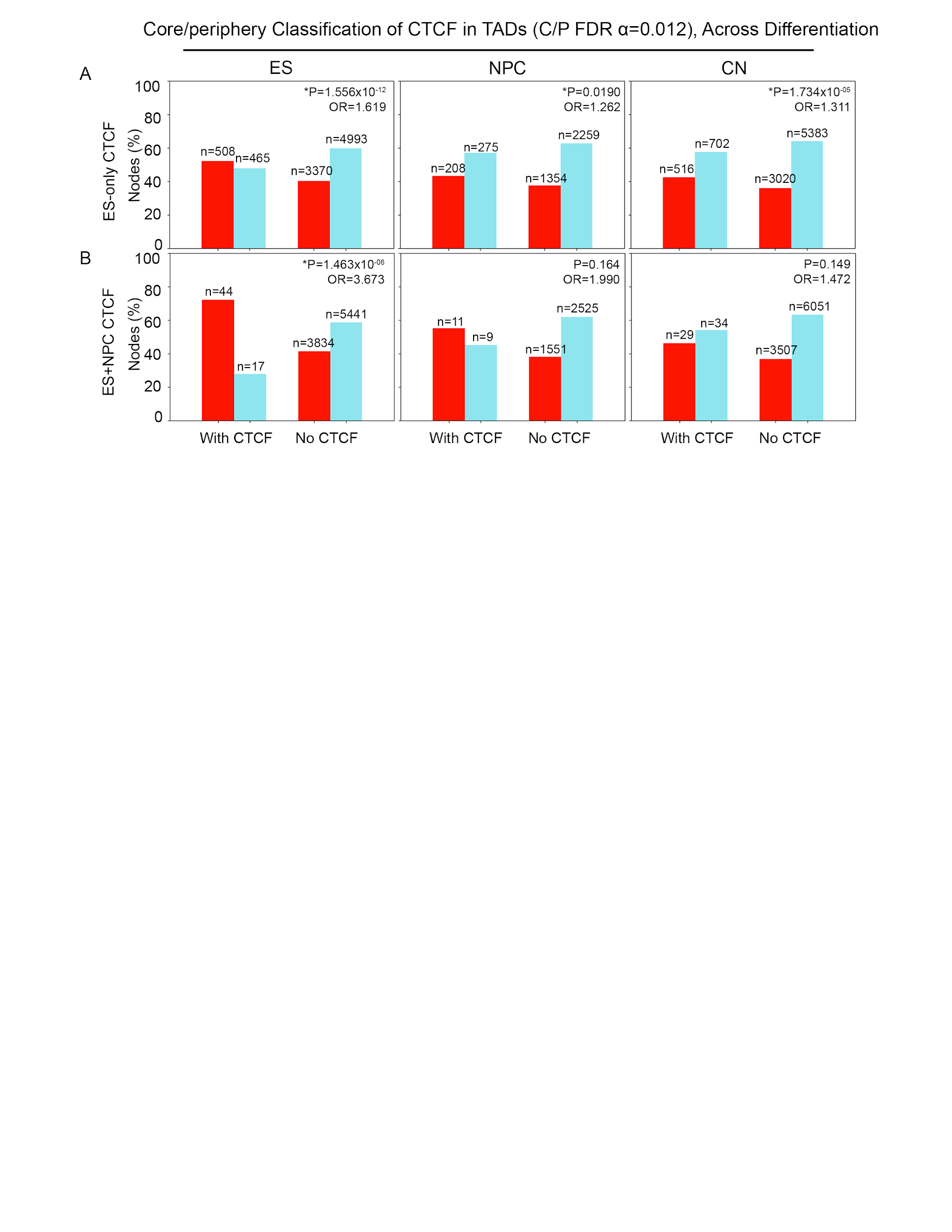 |
| --- |
| **Supplementary Figure 7. Core-Periphery proportions of CTCF occupancy classes not in Figure 4.** (A) Proportions of core and periphery nodes colocalized with ES-only CTCF occupied sites in TADs (core-periphery TAD FDR α=0.012), compared to core and periphery node proportions of unannotated nodes in the same TADs across cell types. Significantly different ES-specific versus unannotated core and periphery proportions are marked with an asterisk. (B) Core and periphery node proportions for ES+NPC CTCF. |

| 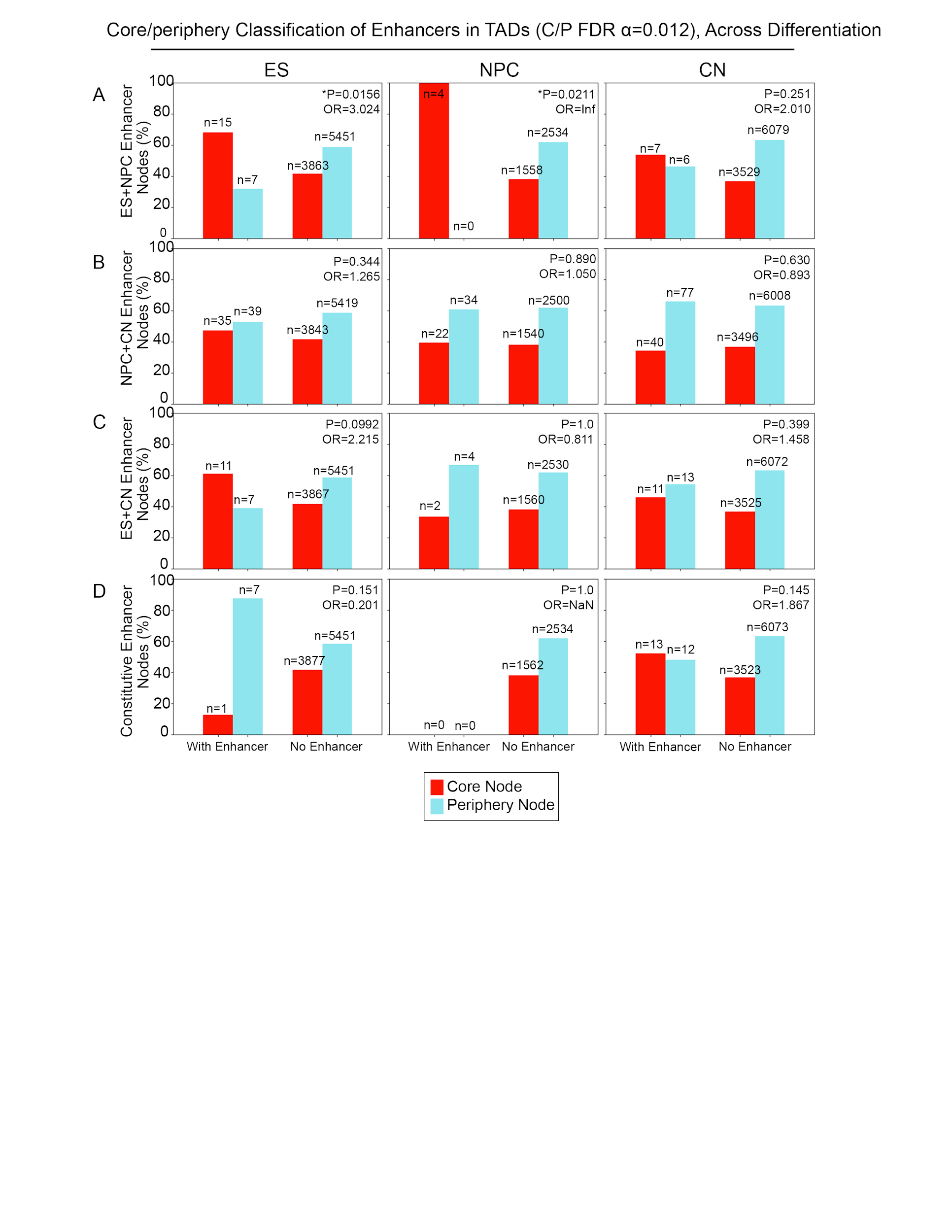 |
| --- |
| **Supplementary Figure 8. Core-Periphery classifications of cell type-specific enhancer cell types not in Figure 4.** (A) Proportions of core and periphery nodes with ES+NPC enhancers in TADs (core-periphery TAD FDR α=0.012), compared to core and periphery node proportions of unannotated nodes in the same TADs across cell types. Significantly different enhancer vs. unannotated core and periphery proportions are marked with an asterisk. (B-D) Core and periphery node proportions for (B) NPC+ES enhancers, (C) ES+CN enhancers, and (D) constitutive enhancers. |

| 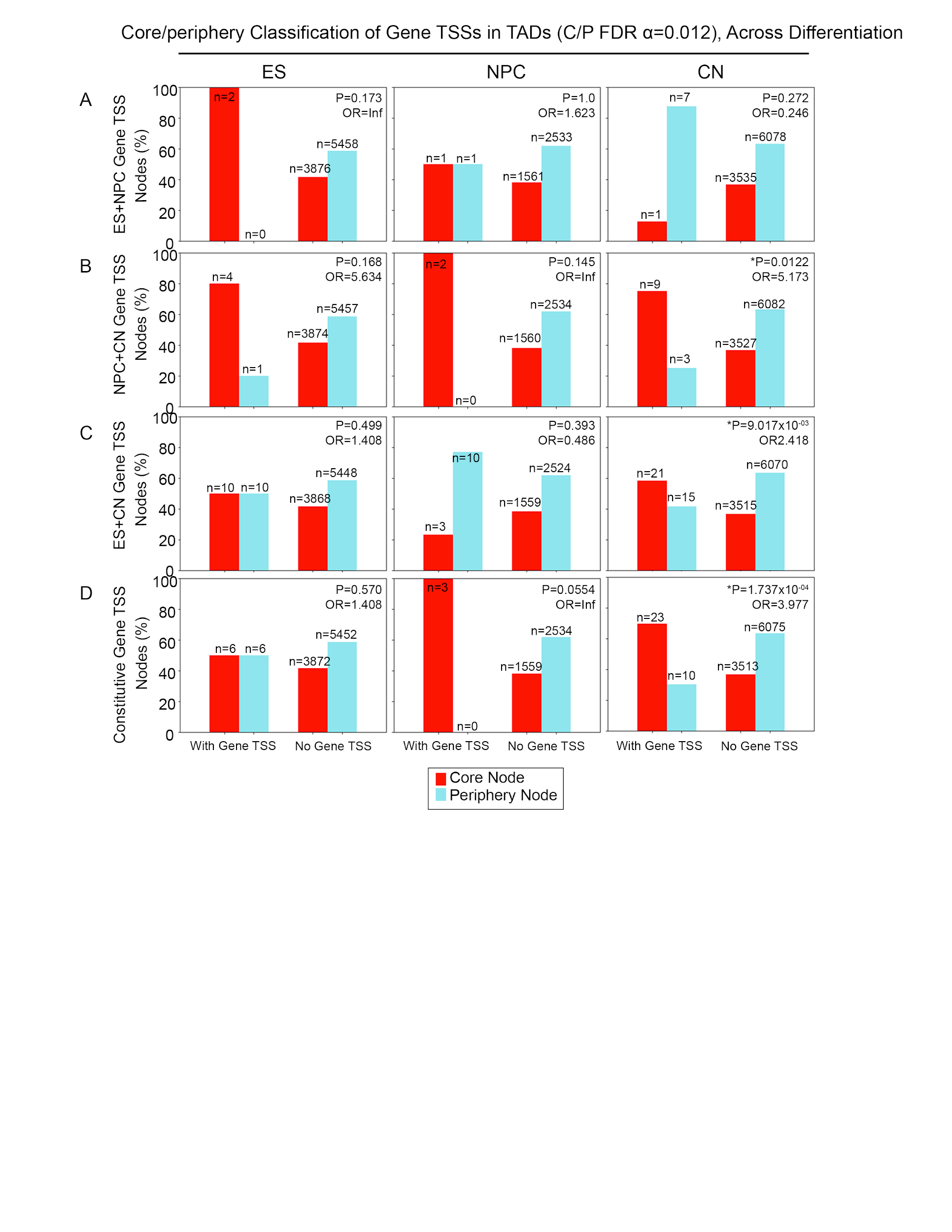 |
| --- |
| **Supplementary Figure 9. Core-Periphery proportions of additional TSS activity classes not in Figure 4.** (A) Proportions of core and periphery nodes colocalized with ES+NPC expressed TSSs in TADs (core-periphery TAD FDR α=0.012), compared to core and periphery node proportions of unannotated nodes in the same TADs across cell types. Significantly different ES+NPC expressed TSSs versus unannotated core and periphery proportions are marked with an asterisk. (B-D) Core and periphery node proportions for (B) NPC+CN expressed TSSs, (C) ES+CN expressed TSSs, (D) constitutively expressed TSSs. |

| 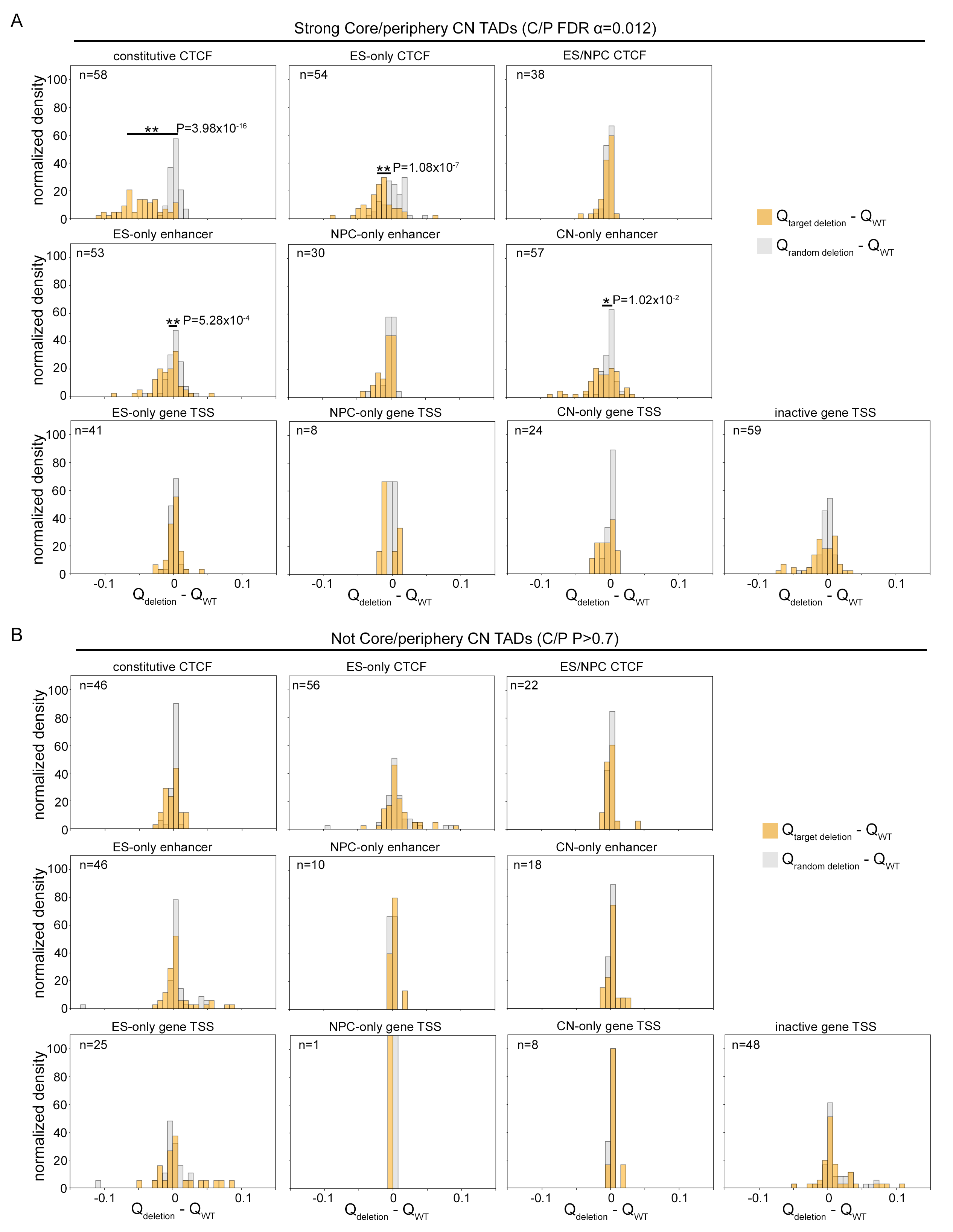 |
| --- |
| **Supplementary Figure 10. Removal of CTCF nodes in strong Core-Periphery behavior CN TADs results in significant decrease in Q compared to removal of nodes at random.** (A) Difference in Q before and after node deletion in strong core-periphery behavior CN TADs (core-periphery FDR α=0.012) for three classes of CTCF (top), three classes of enhancers (middle), and four classes of gene TSS (bottom). Deletion of nodes that intersect or are immediately adjacent to target annotations is compared to deletion of the same number of nodes at random in each TAD. Single asterisk indicates significant difference in distribution means (Mann-Whitney U P<0.05), and double asterisk indicates highly significant difference in distribution means (Mann-Whitney U P<0.005). (B) Difference in Q before and after node deletion in not core-periphery CN TADs (pivalue<0.7) for three classes of CTCF (top), three classes of enhancers (middle), and four classes of gene TSS (bottom). Deletion of nodes that intersect or are immediately adjacent to target annotations is compared to deletion of the same number of nodes at random in each TAD. No significant difference in distribution means was found (Mann-Whitney U, α=0.05). |

| 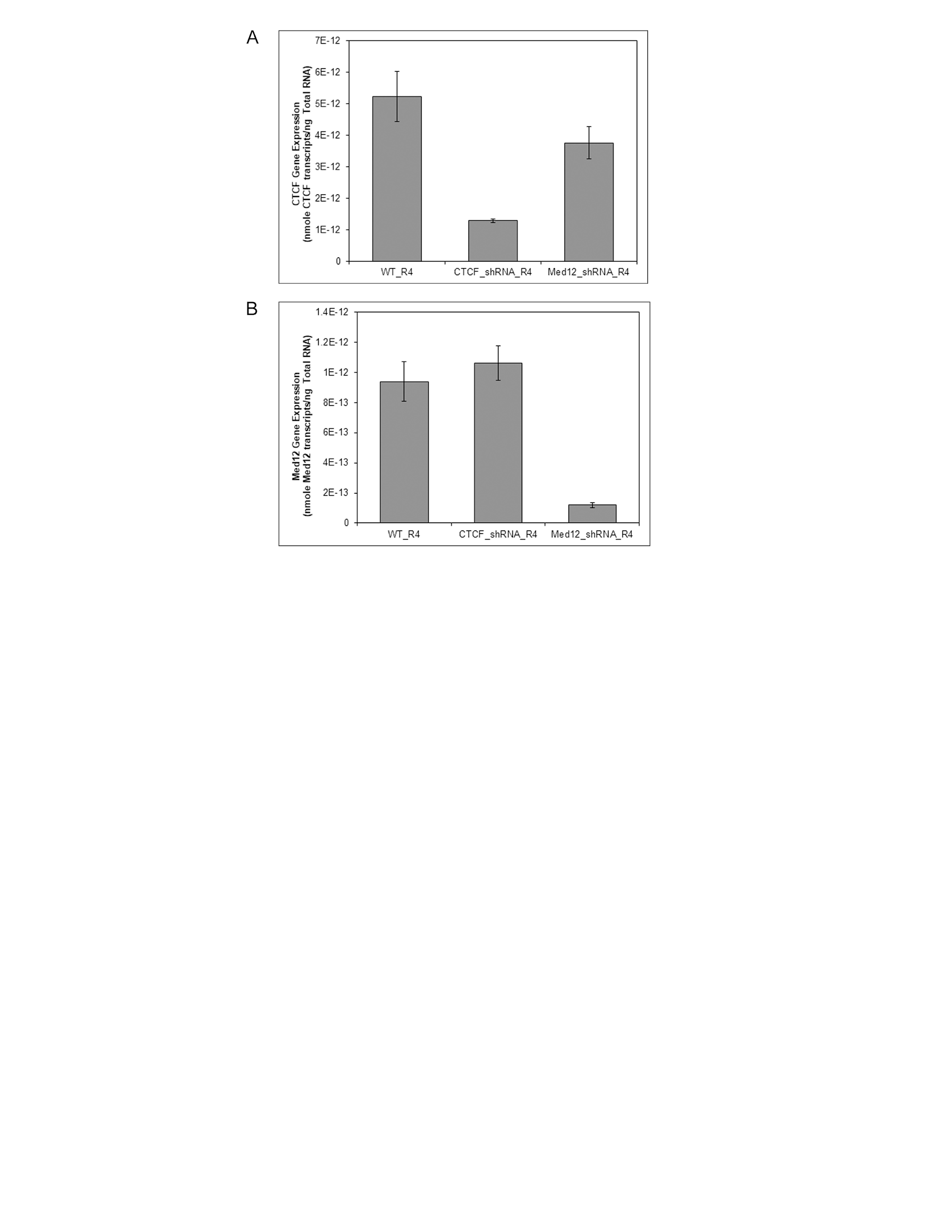 |
| --- |
| **Supplementary Figure 11. CTCF knockdown and Mediator knockdown show reduced target protein knockdown gene expression.** (A) qRT-PCR of CTCF knockdown by shRNA shows reduced gene expression of CTCF mRNA compared to wild type (WT) and Mediator (Med). (B) qRT-PCR of Mediator knockdown by shRNA shows reduced gene expression of Mediator mRNA compared to WT and Med. |


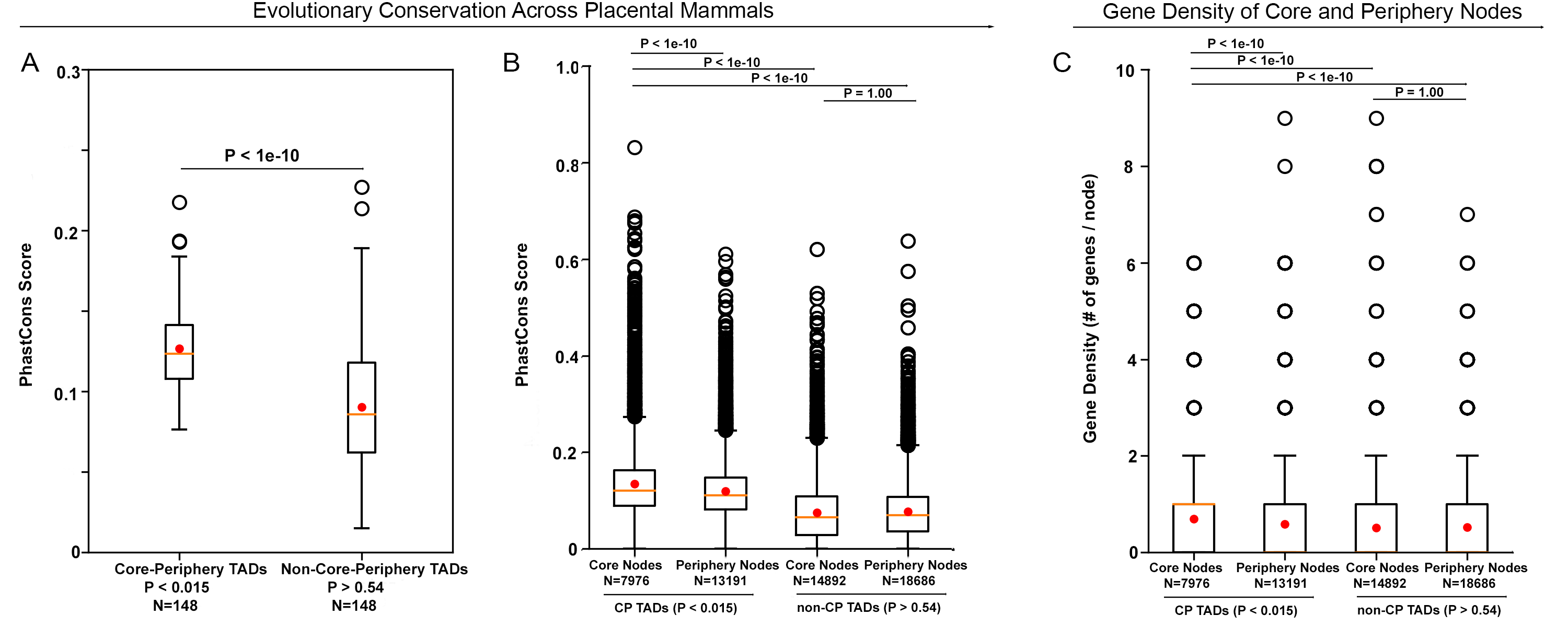


**Supplementary Figure 12. Core-Periphery network structure in cortical neurons is evolutionarily conserved.** (A) Evolutionary conservation in TADs with strong core-periphery structure and TADs devoid of such structure. Pvalue, One-way Mann Whitney U Test. (B) Evolutionary conservation of genomic regions spanning core versus periphery nodes in TADs with strong core-periphery structure and TADs devoid of such structure. Pvalue, One-way Mann Whitney U Test. (C) Gene densities of genomic regions spanning core versus periphery nodes in TADs with strong core-periphery structure and TADs devoid of such structure. Core nodes in core-periphery TADs have significantly higher gene densities than periphery nodes in core-periphery TADs, core nodes in non-core-periphery TADs, and periphery nodes in non-core-periphery TADs. Core nodes in non-core-periphery TADs have similar gene densities to periphery nodes in non-core-periphery TADs. Pvalue, One-way Mann Whitney U Test.

**SUPPLEMENTAL TABLES**

**Table S1: Summary of publically available Hi-C datasets analyzed**

| Cell Type* | Total Mapped Reads (Paired End 1) | Total Mapped Reads (Paired End 2) | Test Hi-C Reference | Test Sample GEO ID |
| --- | --- | --- | --- | --- |
| ES Rep 1 | 2459332626 | 2412188707 | (Bonev et al. 2017) | GSM2533818 |
| ES Rep 2 | 1264525077 | 1229452206 | (Bonev et al. 2017) | GSM2533819 |
| ES Rep 3 | 2676680647 | 2643252421 | (Bonev et al. 2017) | GSM2533820 |
| ES Rep 4 | 508891587 | 500358336 | (Bonev et al. 2017) | GSM2533821 |
| NPC Rep 1 | 1225708794 | 1217162125 | (Bonev et al. 2017) | GSM2533822 |
| NPC Rep 2 | 2078738024 | 2075565501 | (Bonev et al. 2017) | GSM2533823 |
| NPC Rep 3 | 1446791415 | 1439739185 | (Bonev et al. 2017) | GSM2533824 |
| NPC Rep 4 | 3541473074 | 3536875588 | (Bonev et al. 2017) | GSM2533825 |
| CN Rep 1 | 742940091 | 739250691 | (Bonev et al. 2017) | GSM2533826 |
| CN Rep 2 | 742219361 | 737027934 | (Bonev et al. 2017) | GSM2533827 |
| CN Rep 3 | 1051626424 | 1053586132 | (Bonev et al. 2017) | GSM2533828 |
| CN Rep 4 | 1670033598 | 1664066417 | (Bonev et al. 2017) | GSM2533829 |
| CN Rep 5 | 2326750683 | 2094240068 | (Bonev et al. 2017) | GSM2533830 |
| CN Rep 6 | 1798955262 | 1794132818 | (Bonev et al. 2017) | GSM2533831 |

* Footnote: Reps were subsequently merged for analysis

**Table S2: ES DI300 TAD Calls**

Provided in separate spreadsheet.

**Table S3: NPC DI300 TAD Calls**

Provided in separate spreadsheet.

**Table S4: CN DI300 TAD Calls**

Provided in separate spreadsheet.

**Table S5: ES Bad Domain Calls**

Provided in separate spreadsheet.

**Table S6: NPC Bad Domain Calls**

Provided in separate spreadsheet.

**Table S7: CN Bad Domain Calls**

Provided in separate spreadsheet.

**Table S8: Summary of publically available ChIP-seq datasets analyzed**

| Target Protein | Cell Type | Mapped Test ChIP-seq reads (after downsampling)* | Test ChIP Reference | Test Sample GEO ID | Control Samples | Mapped Test ChIP-seq reads (after downsampling)* | Control ChIP Reference | Control Sample GEO ID |
| --- | --- | --- | --- | --- | --- | --- | --- | --- |
| CTCF Rep 1 | mES (E14TG2a) | 28000023 | (Bonev et al. 2017) | GSM2533853 | E14TG2a  Whole Cell Extract | 20000023 | (Bonev et al. 2017) | GSM2533857 |
| CTCF Rep 2 |  |  |  | GSM2533854 |  |  |  |  |
| CTCF Rep 1 | ES-derived NPC | 28000023 | (Bonev et al. 2017) | GSM2533858 | NPC Whole Cell Extract | 20000023 | (Bonev et al. 2017) | GSM2533874 |
| CTCF Rep 2 |  |  |  | GSM2533859 |  |  |  | GSM2533875 |
| CTCF Rep 1 | ES-derived CN | 28000023 | (Bonev et al. 2017) | GSM2533876 | CN Whole Cell Extract | 20000023 | (Bonev et al. 2017) | GSM2533892 |
| CTCF Rep 2 |  |  |  | GSM2533877 |  |  |  | GSM2533893 |
| H3K27ac Rep 1 | mES (v6.5) | 20000023 | (Creyghton et al. 2010) | GSM594578 | C57BL/6  Whole Cell Extract | 20000023 | (Stadler et al. 2011) | GSM747545 |
| H3K27ac Rep 2 |  |  |  | GSM594579 |  |  |  | GSM747546 |
| H3K27ac Rep 1 | ES-derived NPC | 20000023 | (Bonev et al. 2017) | GSM2533868 | NPC Whole Cell Extract | 20000023 | (Bonev et al. 2017) | GSM2533874 |
| H3K27ac Rep 2 |  |  |  | GSM2533869 |  |  |  | GSM2533875 |
| H3K27ac Rep 1 | ES-derived CN | 20000023 | (Bonev et al. 2017) | GSM2533886 | CN Whole Cell Extract | 20000023 | (Bonev et al. 2017) | GSM2533892 |
| H3K27ac Rep 2 |  |  |  | GSM2533887 |  |  |  | GSM2533893 |

* Footnote: Reps were subsequently merged for analysis

**Table S9: UCSC Gene TSS Annotations, 2kb upstream + 2kb downstream**

Provided in separate spreadsheet.

**Table S10: CTCF Peaks present only in the ES cell state (classified as ‘ES-only’)**

Provided in separate spreadsheet.

**Table S11: CTCF Peaks present in the ES and NPC cell states (classified as ‘ES+NPC’)**

Provided in separate spreadsheet.

**Table S12: CTCF Peaks present in the ES, NPC, and CN cell states (classified as ‘constitutive’)**

Provided in separate spreadsheet.

**Table S13: CTCF Peaks present in no cell states (classified as ‘unclassified’)**

Provided in separate spreadsheet.

**Table S14: Enhancer Peaks present only in the ES cell state (classified as ‘ES-only’)**

Provided in separate spreadsheet.

**Table S15: Enhancer Peaks present only in the NPC cell state (classified as ‘NPC-only’)**

Provided in separate spreadsheet.

**Table S16: Enhancer Peaks present only in the CN cell state (classified as ‘CN-only’)**

Provided in separate spreadsheet.

**Table S17: Enhancer Peaks present in the ES and NPC cell states (classified as ‘ES+NPC’)**

Provided in separate spreadsheet.

**Table S18: Enhancer Peaks present in the NPC and CN cell states (classified as ‘NPC+CN’)**

Provided in separate spreadsheet.

**Table S19: Enhancer Peaks present in the ES and CN cell states (classified as ‘ES+CN’)**

Provided in separate spreadsheet.

**Table S20: Enhancer Peaks present in the ES, NPC, and CN cell states (classified as ‘constitutive’)**

Provided in separate spreadsheet.

**Table S21: Enhancer Peaks present in no cell states (classified as ‘unclassified’)**

Provided in separate spreadsheet.

**Table S22: Gene TSS Peaks present only in the ES cell state (classified as ‘ES-only’)**

Provided in separate spreadsheet.

**Table S23: Gene TSS Peaks present only in the NPC cell state (classified as ‘NPC-only’)**

Provided in separate spreadsheet.

**Table S24: Gene TSS Peaks present only in the CN cell state (classified as ‘CN-only’)**

Provided in separate spreadsheet.

**Table S25: Gene TSS Peaks present in the ES and NPC cell states (classified as ‘ES+NPC’)**

Provided in separate spreadsheet.

**Table S26: Gene TSS Peaks present in the NPC and CN cell states (classified as ‘NPC+CN’)**

Provided in separate spreadsheet.

**Table S27: Gene TSS Peaks present in the ES and CN cell states (classified as ‘ES+CN’)**

Provided in separate spreadsheet.

**Table S28: Gene TSS Peaks present in the ES, NPC, and CN cell states (classified as ‘constitutive’)**

Provided in separate spreadsheet.

**Table S29: Gene TSS Peaks present in no cell states (classified as ‘inactive’)**

Provided in separate spreadsheet.

**Table S30: Percent of Annotations in Core and Periphery Nodes in significantly core-periphery TADs from ES cells**

|  | Core Nodes | | | Periphery Nodes | | |
| --- | --- | --- | --- | --- | --- | --- |
|  | CTCF (%) | Enhancer (%) | Gene TSS (%) | CTCF (%) | Enhancer (%) | Gene TSS (%) |
| ES-only | 12.361 | 2.418 | 0.617 | 8.018 | 3.408 | 0.305 |
| NPC-only | 0.000 | 0.592 | 0.025 | 0.000 | 0.951 | 0.000 |
| CN-only | 0.000 | 3.948 | 0.246 | 0.000 | 3.211 | 0.269 |
| ES+NPC | 1.036 | 0.271 | 0.000 | 0.269 | 0.090 | 0.000 |
| NPC+CN | 0.000 | 0.716 | 0.049 | 0.000 | 0.574 | 0.018 |
| ES+CN | 0.000 | 0.222 | 0.197 | 0.000 | 0.108 | 0.108 |
| Constitutive | 4.367 | 0.025 | 0.123 | 0.466 | 0.072 | 0.090 |
| Inactive | N/A | N/A | 2.541 | N/A | N/A | 2.780 |

**Table S31: Percent of Annotations in Core and Periphery Nodes in significantly core-periphery TADs from NPCs**

|  | Core Nodes | | | Periphery Nodes | | |
| --- | --- | --- | --- | --- | --- | --- |
|  | CTCF (%) | Enhancer (%) | Gene TSS (%) | CTCF (%) | Enhancer (%) | Gene TSS (%) |
| ES-only | 12.138 | 3.804 | 0.906 | 10.184 | 2.575 | 0.231 |
| NPC-only | 0.000 | 1.147 | 0.060 | 0.000 | 0.346 | 0.077 |
| CN-only | 0.000 | 5.314 | 0.483 | 0.000 | 3.958 | 0.231 |
| ES+NPC | 0.664 | 0.181 | 0.060 | 0.307 | 0.000 | 0.000 |
| NPC+CN | 0.000 | 1.147 | 0.060 | 0.000 | 0.961 | 0.000 |
| ES+CN | 0.000 | 0.000 | 0.060 | 0.000 | 0.154 | 0.231 |
| Constitutive | 5.556 | 0.000 | 0.121 | 0.730 | 0.000 | 0.000 |
| Inactive | N/A | N/A | 2.657 | N/A | N/A | 3.267 |

**Table S32: Percent of Annotations in Core and Periphery Nodes in significantly core-periphery TADs from cortical neurons**

|  | Core Nodes | | | Periphery Nodes | | |
| --- | --- | --- | --- | --- | --- | --- |
|  | CTCF (%) | Enhancer (%) | Gene TSS (%) | CTCF (%) | Enhancer (%) | Gene TSS (%) |
| ES-only | 13.126 | 3.848 | 0.896 | 10.606 | 3.084 | 0.636 |
| NPC-only | 0.000 | 1.186 | 0.211 | 0.000 | 0.493 | 0.016 |
| CN-only | 0.000 | 5.219 | 0.501 | 0.000 | 4.071 | 0.254 |
| ES+NPC | 0.738 | 0.158 | 0.026 | 0.493 | 0.064 | 0.095 |
| NPC+CN | 0.000 | 0.896 | 0.158 | 0.000 | 0.906 | 0.048 |
| ES+CN | 0.000 | 0.211 | 0.395 | 0.000 | 0.143 | 0.143 |
| Constitutive | 6.800 | 0.264 | 0.448 | 0.636 | 0.127 | 0.111 |
| Inactive | N/A | N/A | 3.795 | N/A | N/A | 3.705 |

**Table S33: Summary of new 5C datasets provided in this study**

| Condition | Cell Type | Total Mapped Reads (Paired End 1) | Total Mapped Reads (Paired End 2) |
| --- | --- | --- | --- |
| WT Rep 1 | ES | 13018770 | 13124851 |
| WT Rep 2 | ES | 12508941 | 12551409 |
| CTCF-KO Rep 1 | ES | 16807941 | 16743382 |
| CTCF-KO Rep 2 | ES | 8064289 | 7973867 |
| Med-KO Rep 1 | ES | 9488654 | 9611543 |
| Med-KO Rep 2 | ES | 12590944 | 12432964 |

**SUPPLEMENTAL METHODS**

**Hi-C Analysis**

**Hi-C Processing**

Raw Hi-C reads from mouse ES cells, NPCs, and CNs generated from Bonev et al. were downloaded from GEO ^1^ (**Supplementary Table 1**). Raw reads were processed with the Hi-C read alignment procedure detailed previously ^2^. In brief, paired-end reads were aligned to mm10 mouse genome using Bowtie2 ^3^ through the HiC-Pro software ^4^. Custom scripts were written to perform matrix assembly, and replicates were merged in this step. Hi-C contact maps were generated at 10kb matrix resolution and balanced using the Knight-Ruiz matrix balancing algorithm.

**Hi-C Poorly-mapped Region Identification**

We first removed genomic regions with poor mapability by instituting a sliding window with size 50 bins and step 25 bins across each Relative Interaction Frequency (Observed) interaction matrix. In each window, bins in which > 25% of possible interactions had counts < 1.0 were flagged as poorly mapped. Windows were removed from downstream analysis if containing >5 flagged bins or a stretch of > 3 consecutive flagged bins (**Supplementary Tables 5-7**). Telomeres and centromeres were also excluded from further analysis.

**Topologically Associated Domain Detection**

Topologically associated domains (TADs) were identified using the directionality index as defined by Ren and colleagues with a DI window of 300 (**Supplementary Tables 2-4**). To ensure that TAD boundary interactions would be captured in calculation, five bins (50kb) were added at the start and end coordinate of every detected TAD. TADs that intersected poorly-mapped, telomeric, or centromeric regions were excluded from consideration (**Supplementary Tables 5-7**).

**Expected distance-dependence and local TAD/subTAD background modeling**

To identify long-range interactions enriched above the expected background signal at each pixel, we modeled the distance-dependent background interactions and the local TAD/subTAD structure for every pixel in the genome. For each observed bin-bin interaction, we computed the donut and lower-left filters as previously described ^5, 6^. We performed sweeps of the p and w parameters and selected 3 and 12, respectively (**Supplementary Figure 2**). We normalized our matrix balanced interaction frequency data by the max value of either the Donut or the lower left filter for every pixel in the genome (**Supplementary Figure 1**), with each pixel assigned a log2(Observed/Expected) value as a distance-dependence and local TAD-corrected interaction frequency. We conducted all downstream network analyses on the log2(Obs/Exp) values in each TAD after thresholding for only the positive values and assigning all negative values nan.

**Network construction on a per TAD basis**

Non-negative log2(Observed/Expected) counts for pixels within valid TADs were used to construct weighted, undirected networks. Nodes represent genomic bins and edges represent the log2(Observed/Expected) interactions between any given pair of nodes. Each node represents an adjacent bin (i.e. genomic interval) on the linear mouse genome.

**Quantifying core-periphery network structure within TADs**

We used a locally greedy Kernighan-Lin graph partitioning algorithm to measure the strength of core-periphery structure in each TAD network and to classify each network node as either core or periphery ^7^. The code that we employed was a Python translation of the core/periphery function provided in the MATLAB Brain Connectivity Toolbox (<https://sites.google.com/site/bctnet/>). Briefly, this algorithm maximizes a core-periphery test statistic, $Q$, by maximizing the strength of intra-core connectivity and minimizing the strength of intra-periphery connectivity. First, we removed any self-loops (diagonal entries) that existed in the network. We then shifted all remaining edge weights in the network by a constant according to **Equation** **1**:

$$\begin{aligned} b_{i,j}=w_{i,j}-\gamma\frac{\sum_{i,j,i\neq j}^{n,n} w_{i,j}}{n\left( n-1 \right)} \left( \boldsymbol{1} \right) \end{aligned}$$

where $w_{i,j}$ is the edge weight between nodes $i$ and $j$, $n$ is the number of nodes in the network, and $\gamma$ is a parameter that weighs the importance of minimizing intra-periphery connections relative to maximizing intra-core connections. When $\gamma$ is 1, both computations in **Equation 1** are weighed with equal importance, and the mean of all shifted edge weights, $b_{i,j}$, is 0. We then normalized the network edge weights according to **Equation 2**:

$$\begin{aligned} \bar{b}_{i,j}=\frac{b_{i,j}}{\sum_{i,j,i\neq j}^{n,n} \left| b_{i,j} \right|} \left( \boldsymbol{2} \right) \end{aligned}$$

after which the absolute value of all edge weights in the network, $\bar{b}_{i,j}$, sum to 1.0. Finally, the algorithm sought to find the partition of core and periphery nodes that would maximize $Q$, given by **Equation 3**:

$$\begin{aligned} Q=\sum_{i,j,i\neq j}^{n,n} \bar{b}_{i,j} \delta\left( C_{i},C_{j} \right) \left( \boldsymbol{3} \right) \end{aligned}$$

where $C_{i}$ and $C_{j}$ are the core-periphery classifications of nodes $i$ and $j$, and $\delta\left( C_{i},C_{j} \right)$ is equal to 1 if both $C_{i}$ and $C_{j}$ are core, -1 if both $C_{i}$ and $C_{j}$ are periphery, and 0 if $C_{i}$ and $C_{j}$ are different. $Q$ takes on a minimum possible value of -1 and a maximum possible value of 1.

The maximization algorithm is briefly described below. Each node in the network is first given a core or periphery classification at random, and an initial $Q$ is calculated. The algorithm then makes passes until the optimal core/periphery classification for all nodes is found. Each pass proceeds as follows. We first calculate a list of hypothetical $Q$s equal to the number of nodes in the network, where each value in the list is the $Q$ recomputed when the core/periphery classification of a single node is reversed. We then actually reverse the core/periphery classification of the node that gave the highest hypothetical $Q$, and repeat this process. Once a node classification has been reversed, it can no longer be reversed again in the same pass. We proceed to reverse the core/periphery classification of each node, until all nodes have been reversed exactly once. The intermediate core/periphery classification, $C$, in the first pass that resulted in the highest overall $Q$ is then used as the starting $C$ for the second pass. The algorithm proceeds with passes until the increase in $Q$ per pass falls under a predetermined convergence threshold. The final $Q$ at the end of all passes is given as the maximized core/periphery statistic, and subsequently defined to be the core/periphery strength of the network. The final $C$ is given as the optimal classification of cores and peripheries. One caveat of this maximization algorithm is that it is heuristic in nature, and thus vulnerable to landing in local minima during the iterative process.

**Hypothesis testing for the existence of core-periphery structure in TADs compared to non-TAD locations in the genome**

Having generated $Q$ for each TAD in all three cell types, we first wished to investigate the overall effect of TAD boundary alignment on core-periphery structure under the following null and alternative hypotheses:

- H_0_: The difference in core/periphery structure strength between genomic windows aligned to TAD boundaries and randomly placed, same size genomic windows is zero.
  $\mathrm{mean}Q$_TADs_genomewide_ = $\mathrm{mean} Q$_randomly_aligned_size_matched_window_genomewide_
- H_A_: The difference in core/periphery structure strength between genomic windows aligned to TAD boundaries and randomly placed, same size genomic windows is not zero.
  $\mathrm{mean}Q$_TADs_genomewide_ ≠ $\mathrm{mean} Q$_randomly_aligned_size_matched_window_genomewide_

In each cell type, we compared the distribution of $Q$ values obtained when genomic windows were aligned to TAD boundaries, to a distribution of $Q$ values obtained when genomic windows were aligned at random. Specifically, this control sample was generated by shifting the alignment of the TAD window at random along the chromosome for each TAD, while preserving window size. A Kolmogorov–Smirnov test was used to determine whether there was a significant difference in distribution between the $Q$ values obtained when genomic windows were aligned to TAD boundaries, and the distribution of $Q$ values obtained when genomic windows were aligned at random (**Figure 2**).

We also repeated this test by using a random network as the control sample instead of a randomly placed, size-matched genomic interval. In this case, the random network control sample was modeled as an Erdos-Renyi graph in which the empirical edge weight distribution was maintained via a process of permuting edge pairs ^8^. The null and alternative hypotheses were:

- H_0_: The difference in core/periphery structure strength between genomic windows aligned to TAD boundaries and a same degree, same strength Erdos-Renyi random graph is zero.
  $\mathrm{mean}Q$_TADs_genomewide_ = $\mathrm{mean} Q$_Erdos-Renyi_
- H_A_: The difference in core/periphery structure strength between genomic windows aligned to TAD boundaries and and a same degree, same strength Erdos-Renyi random graph is not zero.
  $\mathrm{mean}Q$_TADs_genomewide_ ≠ $\mathrm{mean} Q$_Erdos-Renyi_

A Kolmogorov–Smirnov test was used to determine whether there was a significant difference in distribution between the $Q$ values obtained when genomic windows were aligned to TAD boundaries, and the distribution of $Q$ values obtained from Erdos-Renyi random graphs (**Supplementary Figure 3**).

**Hypothesis testing for the degree of core-periphery structure within each individual TAD in each cell type**

We also wished to identifiy the individual TADs in each cell type that exhibit the strongest core-periphery structure. We used the following null and alternative hypotheses:

- H_0_: The core-periphery $Q$ of an individual TAD is not significantly different than the distribution of core-periphery $Q$ from 10,000 randomly placed, same sized windows.
  $Q$_TAD_ = $Q$_random_
- H_A_: The core-periphery $Q$ of an individual TAD is significantly different than the distribution of core-periphery $Q$ from 10,000 randomly placed, same sized windows.

$Q$_TAD_ ≠ $Q$_random_

In each cell type, we compared the $Q$ value from each individual TAD to the distribution of $Q$ values obtained from 10,000 randomly placed, size-matched genomic windows. An empirical p-value was computed as the fraction of the null distribution that was equal to or greater than the test statistic. P-values were corrected for multiple testing by using the Storey-Tibshirani qvalue ^9^. In each cell type, significantly core-periphery TADs were defined as the subset of TADs that pass a false discovery threshold of 1.2%.

**ChIP-seq Analysis**

**Alignments and peakcalling**

Published ChIP-seq data were downloaded from GEO and reanalyzed according to Supplemental Table S8. In brief, reads were aligned to mouse genome build mm10 using Bowtie with default mismatch parameters . After removing optical and PCR duplicates, mapped reads were downsampled to equal read numbers across cellular states. CTCF ChIP-seq libraries for ES, NPC, and CN were downsampled to 28 million unique, non-redundant reads, and Input libraries were downsampled to 20 million unique, non-redundant reads. H3K27ac ChIP-seq libraries for ES, NPC, and CN were downsampled to 20 million reads, and Input libraries were downsampled to 20 million unique, non-redundant reads. Peaks were called using Model-based Analysis for ChIP Sequencing v2.0 (MACS2) ^10-12^. For CTCF ChIP-seq, a p-value cutoff of P < 1x10^-4^ was used. For H3K27ac ChIP-seq, a p-value cutoff of P < 1x10^-8^ and a broad peak p-value cutoff of P < 1x10^-4^ were used.

**Enhancer, TSS, and CTCF Annotation parsing**

Enhancer, transcription start site (TSS), and CTCF sites were each parsed into seven annotation classes: ES-only, NPC-only, CN-only, ES+NPC, NPC+CN, ES+CN, and constitutive. TSS annotations were further parsed into an eighth additional class: inactive.

The following procedure was used to parse enhancer classes: H3K27ac peaks in each of the three cell types were first subtracted for any that were within 2kb of transcription start sites (TSS). The peaks were then merged across the three cell types, with those within 6kb combined into a single peak. For each merged peak, we found the mean H3K27ac IP pileup signal in each cell type, and divided that by the matching mean input pileup signal. We then defined an IP/input activation threshold in each cell type as the 1st percentile mean H3K27ac IP/input value of unmerged H3K27ac peaks in the cell type of interest, after subtraction for those that were within 2kb of a TSS. ES-only enhancer peaks were defined as merged peaks with a mean IP/input value at least twice as high in ES cell than in NPC or CN, and which was greater than or equal to the ES activation threshold. NPC-only and CN-only enhancer peaks were parsed in a similar way. ES+NPC enhancer peaks were defined as merged peaks with a mean IP/input value at least twice as high in ES cell and in NPC cell type than in CN, but less than a two-fold difference between ES and NPC. Furthermore, the mean IP/input values in ES and NPC were required to be greater than or equal to the respective cell type activation thresholds, but the IP/input value in CN was required to be less than the CN activation threshold. NPC+CN and ES+CN enhancer peaks were parsed in a similar way. Finally, constitutive enhancers were defined as merged peaks with less than two-fold difference in mean IP/input value across all three possible cell type pairs, each of which greater than or equal to the respective cell type activation threshold.

The following procedure was used to parsed TSS classes. H3K27ac peaks in each of the three cell types that were within 2kb of TSS were merged together, with those within 4kb combined into a single peak. For each merged peak, we found the mean H3K27ac IP pileup signal in each cell type, and divided that by the matching mean input pileup signal. We then defined an IP/input activation threshold in each cell type as the 1st percentile mean H3K27ac IP/input value of unmerged H3K27ac peaks that were within 2kb of a TSS in the cell type of interest. ES-only peaks were defined as merged peaks with a mean IP/input value at least twice as high in ES cell type than in NPC or CN, and which was greater than or equal to the ES activation threshold. ES-only TSS annotations were then defined as all TSS-2kb to TSS+2kb regions that intersected ES-only peaks. NPC-only and CN-only TSS annotations were parsed in a similar way. ES+NPC peaks were defined as merged peaks with a mean IP/input value at least twice as high in ES cell type and in NPC cell type than in CN, but less than a two-fold difference between ES and NPC. Furthermore, the mean IP/input values in ES and NPC were required to be greater than or equal to the respective cell type activation thresholds, but the IP/input value in CN was required to be less than the CN activation threshold. ES+NPC TSS annotations were then defined as all TSS-2kb to TSS+2kb regions that intersected ES+NPC peaks. NPC+CN and ES+CN TSS annotations were parsed in a similar way. Constitutive peaks were defined as merged peaks with less than two-fold difference in mean IP/input value across all three possible cell type pairs, each of which greater than or equal to the respective cell type activation threshold. Constitutive TSS annotations were defined as all TSS-2kb to TSS+2kb regions that intersected constitutive peaks. Finally, inactive TSS annotations were defined as TSS-2kb to TSS+2kb regions that did not intersect H3K27ac peaks in any of the three cell types.

The following procedure was used to parse CTCF classes. CTCF peaks in each of the three cell types were first merged together, with overlapping peaks combined into a single peak. For each merged peak, we found the mean CTCF IP pileup signal in each cell type, and divided that by the matching mean input pileup signal. We then defined an IP/input activation threshold in each cell type as the 1st percentile mean CTCF IP/input value of unmerged CTCF peaks in the cell type of interest. ES-only CTCF peaks were defined as merged peaks with a mean IP/input value at least five times as high in ES cell than in NPC or CN, and which was greater than or equal to the ES activation threshold. NPC-only and CN-only CTCF peaks were parsed in a similar way. ES+NPC CTCF peaks were defined as merged peaks with a mean IP/input value at least five times as high in ES cell and in NPC than in CN, but less than a five-fold difference between ES and NPC. Furthermore, the mean IP/input values in ES and NPC were required to be greater than or equal to the respective cell type activation thresholds, but the IP/input value in CN was required to be less than the CN activation threshold. NPC+CN and ES+CN enhancer peaks were parsed in a similar way. Finally, constitutive CTCF peaks were defined as merged peaks with less than five-fold difference in mean IP/input value across all three possible cell type pairs, each of which greater than or equal to the respective cell type activation threshold.

**Core-Periphery Annotation Intersection Analysis**

For each cell type, core and periphery nodes in significantly core-periphery TADs were each separated into those that intersected annotations and those that did not. We summed the number of nodes that intersected each annotation type, and then removed those nodes from further consideration. The order of annotation analysis was based on observed importance and abundance. The order was as follows: constitutive CTCF, ES-only CTCF, ES+NPC CTCF, ES-only enhancer, NPC-only enhancer, CN-only enhancer, ES+NPC enhancer, NPC+CN enhancer, ES+CN enhancer, constitutive enhancer, ES-only gene TSS, NPC-only gene TSS, CN-only gene TSS, ES+NPC gene TSS, NPC+CN gene TSS, ES+CN gene TSS, constitutive gene TSS, and inactive gene TSS. No peaks were reported for NPC-only CTCF, CN-only CTCF, NPC+CN CTCF, and ES+CN CTCF, so those annotations were not considered in the analysis. Numbers of annotations were plotted as percentages for core nodes and periphery nodes.

**Core-Periphery Annotation Enrichment Analysis**

We compared the number of annotated core nodes and periphery nodes to the number of unannotated core nodes and periphery nodes in significantly core-periphery TADs. To find differences between annotated nodes and unannotated nodes, we conducted a hypothesis test under the following null and alternative hypotheses:

- H_0_: The ratio of annotated core nodes to annotated periphery nodes is the same as the ratio of unannotated core nodes to unannotated periphery nodes.
- H_a_: The ratio of annotated core nodes to annotated periphery nodes is different than the ratio of unannotated core nodes to unannotated periphery nodes.

We considered in turn each class of CTCF, enhancer, and TSS annotations across ES, NPC, and CN cells. In each case, Fisher’s Exact test p-values and odds ratios were reported.

***In silico* Mutagenesis**

To test the effect of annotation removal on TAD core-periphery structure, we devised a test that simulates in silico mutagenesis. We worked on the assumption that if an annotation type is essential for the classification of core vs. periphery nodes, then the removal of the annotated nodes should result in an overall decrease in core-periphery structure relative to removal of nodes at random. We tested each annotation of interest under the following null and alternative hypotheses:

- H_0_: $Q$ changes by the same amount when TADs are subjected to annotated node deletion, compared to when they are subjected to random node deletion.
  $Q$_diff, annotated_ = $Q$_diff, random_
- H_a_: $Q$ changes by a different amount when TADs are subjected to annotated node deletion, compared to when they are subjected to random node deletion.
  $Q$_diff, annotated_ = $Q$_diff, random_

To test this hypothesis, we first isolated significantly core-periphery TADs (FDR ⍺=0.012) in one cell type that intersected at least one instance of the annotation of interest. We then deleted all nodes within the TAD that directly intersected the annotation of interest, or are immediately adjacent to directly-intersected nodes, and computed the change in $Q$ for each TAD after deletion. TADs in which more than 50% of all nodes were deleted were disqualified at this stage. We compared this $Q$_diff_ distribution to a control sample in which $Q$_diff_ was calculated on the same TADs, subjected to deletion in each case of the same number of nodes at random. A two-tailed Mann-Whitney U test with alpha=0.05 was used to indicate significant difference between the two distributions. This hypothesis test was performed in each cell type for the following annotations: constitutive CTCF, ES+NPC CTCF, ES-only CTCF, ES-only enhancers, NPC-only enhancers, CN-only enhancers, ES-only gene TSS, NPC-only gene TSS, CN-only gene TSS, and inactive TSS.

**Placental mammal conservation score in TADs with and without core-periphery structure**

We hypothesized that TADs with stronger core-periphery structure would demonstrate greater evolutionary conservation across species compared to TADs devoid of core-periphery structure. To test our hypothesis, PhastCons placental mammal conservation scores were downloaded from UCSC Genome Browser. TADs were stratified into two bins of core-periphery structure: those less than the 7^th^ percentile (P < 0.015) and those greater than the 93^rd^ percentile (P > 0.54). Next, we plotted the PhastCons score for TADs in the core-periphery group and the non-core-periphery group. We formulated the following null and alternative hypotheses:

- H_0_: The mean conservation scores of TADs in the highest core-periphery p-value bin and the mean conservation scores of TADs in the lowest core-periphery p-value bin belong to the same distribution.
- H_A_: The mean conservation scores of TADs in the highest core-periphery p-value bin and the mean conservation scores of TADs in the lowest core-periphery p-value bin belong to different distributions.

We performed a one-tailed Mann-Whitney U test to compare PhastCons scores (**Supplementary Figure 12A**). We performed additional analyses on gene density and conservation at core versus periphery nodes in strongly core-periphery TADs and TADs devoid of core-periphery structure (**Supplementary Figure 12B-C**).

**Neurodevelopmental disease-associated mutation statistical tests**

Common hg19 variants associated with Schizophrenia (n=108), Autism Spectrum Disorder (n=182), and Obsessive-Compulsive Disorder (n=32) were acquired from the following sources:

- Schizophrenia: Schizophrenia Working Group of the Psychiatric Genomics, *Nature*, 2014 ^13^
- Autism Spectrum Disorder: Autism Spectrum Disorders Working Group of The Psychiatric Genomics, *Mol Autism*, 2017 (European population) ^14^
- Obsessive-Compulsive Disorder: Mettheisen et al., *Mol Psychiatry*, 2015 ^15^

RsIDs for each disease set were uploaded to SNPsnap ^16^ in order to generate 10,000 matched ‘background’ single nucleotide variants (SNVs) for each disease-associated SNV (daSNV). daSNVs were matched according the 1000Genomes Phase 3 European dataset at a linkage disequilibrium (LD) distance cut-off of r^2^=0.7 and LD buddies at r^2^=0.7. daSNVs that could not be background matched using SNPsnap were discarded, thus reducing the daSNV LD blocks for Schizophrenia from n=108 to n=91 and the number of Autism Spectrum Disorder (ASD) daSNV LD blocks from n=182 to n=137, The number of Obsessive-Compulsive Disorder (OCD) daSNV LD blocks remained n=32. Genome-wide LD block r^2^ values for all SNV pairs were downloaded from the SNIPA tool ^17^. For each daSNV and background SNV, an LD block was identified as the set of constituent SNVs linked by r^2^>0.7. Background SNV LD blocks that overlapped each other or a daSNV LD block were removed. daSNVs that fell within exons or gene promoters (2 kb upstream of TSS) were discarded from analysis, thus reducing the number of Schizophrenia daSNV LD blocks from n=91 to n=83, the number of ASD daSNV LD blocks from n=137 to n=128, and the number of OCD LD blocks from n=32 to n=30. All sets of LD blocks were lifted over to the mm10 genome build using the liftOver tool from UCSC genome browser with default parameters. After liftover to mm10, the number of Schizophrenia daSNV LD blocks reduced from n=83 to n=77, the number of ASD daSNV LD blocks reduced from n=128 to n=101, and the number of OCD daSNV LD blocks reduced from n=30 to n=27. Additionally, some lifted daSNV LD blocks were discarded if they spanned more than one chromosome. After filtering, the number of Schizophrenia daSNV LD blocks reduced from n=77 to n=67, the number of ASD daSNV LD blocks reduced from n=101 to n=96, and the number of OCD da SNV LD blocks reduced from n=27 to n=25. The size of each LD block, disease and background, was calculated as the number of constituent SNPs after liftOver.

For each daSNV, 5 background SNVs with the same size LD block were selected. If fewer than 5 background LD blocks of the exact same size existed, background LD blocks of size one greater and one smaller than the daSNV LD block in question were included in the set of 5 size-matched background SNV LD blocks. The size of included background blocks was iteratively increased until 5 size-matched background LD blocks could be selected. If fewer than 5 background SNV LD blocks had a size within 10 of the daSNV LD block, successful background matching could not occur and the process was stopped. daSNVs which could not be successfully matched to 5 background SNV LD blocks were removed from further analysis. Four Schizophrenia daSNV LD blocks were discarded as a result, reducing the total number from n=67 to n=63. If more than 5 background LD blocks were equally able to be matched to a given daSNV, 5 were randomly chosen. We computed 100 bootstrapped sets of background, size-matched LD blocks for each daSNV LD block.

We hypothesized that we would observe a greater frequency of daSNV LD blocks in strongly core-periphery TADs than LD blocks for SNVs not yet associated with the diseases analyzed in this paper. To test this hypothesis, we stratified daSNV (n=184) and background SNV LD blocks (n=920) across Schizophrenia (n=63), ASD (n=96), and OCD (n=25) into those that intersected only significant, strong core-periphery TADs (TADs whose p-values were less than 0.015) and those that did not intersect core-periphery TADs. We constructed a 2x2 contingency table to test the following null and alternative hypotheses:

- H_0_: The relative proportion of daSNV LD blocks that intersect strongly core-periphery CN TADs vs. CN TADs devoid of core-periphery structure is the same as the relative proportion of background SNV LD blocks that intersect strongly core-periphery CN TADs vs. CN TADs devoid of core-periphery structure.
- H_a_: The relative proportion of daSNV LD blocks that intersect strongly core-periphery CN TADs vs. CN TADs devoid of core-periphery structure is not the same as the relative proportion of background SNV LD blocks that intersect strongly core-periphery CN TADs vs. CN TADs devoid of core-periphery structure.

To fill in the contingency table, we counted each intersecting LD block once if at least one constituent SNV intersected a core-periphery or non-core-periphery TAD. We ensured that each category in the contingency table was mutually exclusive. Disease associated and background SNV LD blocks were not counted twice if they intersected both a core-periphery and non-core-periphery TAD. Additionally, background SNV LD blocks were only counted if they intersected TADs not containing daSNVs. Then, we conducted a Fisher’s Exact test with ⍺=0.05 to determine whether a significant difference in relative proportions existed. One hundred Fisher’s Exact tests were carried out across all randomized sets to obtain a median odd’s ratio (OR) and p-value (**Figure 7A**).

We also tested the hypothesis that daSNV LD blocks might have preference to either core or periphery nodes in the CN TAD network. To test this hypothesis, we examined daSNV and background SNV LD blocks intersecting core and periphery nodes within core-periphery TADs only (P < 0.015). For each neuropsychiatric disorder, we examined four mutually exclusive categories: daSNV LD blocks intersecting at least one core node, daSNV LD blocks only intersecting periphery nodes, background SNV blocks intersecting at least one core node, and background SNV LD blocks only intersecting periphery nodes. Using this 2x2 contingency table, we tested the following null and alternative hypotheses:

- H_0_: The relative proportion of daSNV LD blocks is the same in core versus periphery nodes in CN TADs compared to the relative proportion of background SNV LD blocks.
- H_a_: The relative proportion of daSNV LD blocks is not the same in core versus periphery nodes in CN TADs compared to the relative proportion of background SNV LD blocks.

To fill in the contingency table, we counted each intersecting LD block once if at least one constituent SNV intersected a core node. If the LD block did not intersect any core nodes, then the count was assigned to the periphery category. Then, we conducted a Fisher’s Exact test with ⍺=0.05 to determine whether a significant difference in relative proportions existed. One hundred Fisher’s Exact tests were carried out across all randomized sets to obtain a median odd’s ratio (OR) and p-value (**Figure 7B**).

**5C Experimental Procedures**

**ES Cell Culture**

Murine V6.5 ES cells (genotype 129SvJae x C57BL/6; male) were procured from Novus Biologicals at passage 18 and expanded at 37° in 5% CO2 on Mitomycin-C-inactivated MEF feeder layers. ES cell expansion media consisted of Dulbecco’s Modified Eagle Medium (DMEM) supplemented with 15% fetal bovine serum (FBS, GIBCO), 103 U/ml leukemia inhibitory factor (ESGRO, Millipore), 1 × nonessential amino acids (Lonza), 0.1 mM 2-mercaptoethanol, 4 mM L-glutamine (Cellgro), and 1× penicillin/streptomycin (Cellgro). Media was exchanged every 2 days and the cells were passaged at approximately 70% confluence. After initial expansion, ES cells were passaged 1–2 times on 0.1% gelatin to remove contaminating feeder cells. ES cells were ∼70%–75% confluent at the time of fixation for downstream assay.

**qRT-PCR**

mRNA transcripts were quantified using qRT-PCR. Briefly, RNA was extracted from ES cells, stage 3 NPCs, and stage 4 NPCs with an RNeasy Mini kit (QIAGEN Inc, Valencia, CA). Reverse transcription for complementary DNA synthesis was performed with 1 μg of RNA per sample using the SuperScript First Strand Synthesis System for RT-PCR (Invitrogen). Gene expression was assayed with quantitative RT-PCR using the MyIQ cycler (BioRad). Transcript concentrations were calculated using standard curves and normalized to GAPDH expression levels.

**Lentiviral shRNA characterization**

Lentiviral shRNA plasmids cloned into the pLKO.1 vector were purchased from Open Biosystems (Thermo Scientific). The shRNA used in this study is: CTCF shRNA (clone ID: TRCN0000039019, shRNA sequence: ATTACCAACTACTTTCTCTGC). Replication-incompetent lentiviral particles were produced using the Trans-Lentiviral Packaging kit (Open Biosystems) and the calcium phosphate transfection reagent in H293T cells as described (http://www.thermoscientificbio.com/uploadedFiles/Resources/Trans-Lentiviral-Packaging-Technical-Manual.pdf). Virus was harvested by collecting H293T medium, spinning at 500 g for 10 minutes at room temperature, and transferring supernatant to a new 15 mL conical tube. One volume of Lenti-X Concentrator solution (Clontech) was added to three volumes of supernatant, incubated at 4C overnight, and then centrifuged at 1500 g at 4C for 45 minutes to pellet virus particles. Pellet was resuspended in 1 mL DMEM, aliquoted, and stored at -80C until use. Viral titer was calculated as ~107 −108 U/µl using previously described methods (http://mellgenlabs.com/Documents/lentititer_protocol.htm).

**Lentiviral Transductions**

V6.5 Murine ES cells were expanded on 100 mm tissue culture polystyrene petri dishes (Corning, Corning, NY) containing confluent Mitomycin-C treated MEFs. After expansion, ES cells were passaged 1× onto gelatin-coated petri dishes to purify feeders and then seeded at a density of 2 × 106 cells per 100 mm plate. Two days after seeding, ES cell media with 6 μg/ml hexadimethrine bromide (polybrene, Sigma) was added to the cells. After 15 min of incubation at 37°C, lentiviral particles (viral titer of ∼106−107 TU/μl, volume for MOI of 5:1) were gently added to the cells and incubated overnight. The media was then replaced with new ES cell medium for an additional 24 hr. The following day, and for each of the next 3 days, media was exchanged with ES cell media containing 2–3.5 μg/ml puromycin (GIBCO).

**3C and 5C**

3C and 5C were performed exactly as previously described in wild type ES cells and ES cells knocked down for CTCF ^6^.

**5C Analysis**

5C reads were analyzed according to the computational procedures detailed in our previous works ^6, 18-22^. In brief, 5C data were first trimmed for primers that showed severe under-enrichment by observation, and then quantile normalized. 5C data were then balanced via a modified Express matrix balancing algorithm ^21^. 5C data were binned at 4kb resolution. A 20kb smoothing window was applied to reduce spatial noise. Long-range chromatin interactions were extracted by normalizing binned 5C counts using an expected distance-dependent model generated by LOWESS regression and then using a local donut filter as described by Rao et al. ^5^.

We investigated the effect of knock-down of the architectural protein CTCF on core-periphery structure using 5C maps at the Sox2 and Olig1/2 genomic regions (**Supplementary Table 33**). We predicted that the core-periphery test statistic $Q$ would decrease if architectural proteins were perturbed. We isolated 3 subTADs each in the Olig1-Olig2 and Sox2 regions. $Q$ was calculated and compared for each of the subTADs for two wild type replicates and and two replicates of CTCF knockdown.

**Supplementary References**

1. Bonev, B. et al. Multiscale 3D Genome Rewiring during Mouse Neural Development. *Cell* **171**, 557-572 e524 (2017).

2. Hsu, S.C. et al. The BET Protein BRD2 Cooperates with CTCF to Enforce Transcriptional and Architectural Boundaries. *Mol Cell* **66**, 102-116 e107 (2017).

3. Langmead, B. & Salzberg, S.L. Fast gapped-read alignment with Bowtie 2. *Nat Methods* **9**, 357-359 (2012).

4. Servant, N. et al. HiTC: exploration of high-throughput 'C' experiments. *Bioinformatics* **28**, 2843-2844 (2012).

5. Rao, S.S. et al. A 3D map of the human genome at kilobase resolution reveals principles of chromatin looping. *Cell* **159**, 1665-1680 (2014).

6. Beagan, J.A. et al. YY1 and CTCF orchestrate a 3D chromatin looping switch during early neural lineage commitment. *Genome Res* (2017).

7. Kernighan, B.W. & Lin, S. An efficient heuristic procedure for partitioning graphs. *The Bell System Technical Journal* **49**, 291-307 (1970).

8. Paul, E. & Alfréd, R. On Random Graphs I. *Publicationes Mathematicae (Debrecen)* **6** (1959).

9. Storey, J.D. & Tibshirani, R. Statistical significance for genomewide studies. *Proc Natl Acad Sci U S A* **100**, 9440-9445 (2003).

10. Feng, J., Liu, T., Qin, B., Zhang, Y. & Liu, X.S. Identifying ChIP-seq enrichment using MACS. *Nat Protoc* **7**, 1728-1740 (2012).

11. Feng, J., Liu, T. & Zhang, Y. Using MACS to identify peaks from ChIP-Seq data. *Curr Protoc Bioinformatics* **Chapter 2**, Unit 2 14 (2011).

12. Zhang, Y. et al. Model-based analysis of ChIP-Seq (MACS). *Genome Biol* **9**, R137 (2008).

13. Schizophrenia Working Group of the Psychiatric Genomics, C. Biological insights from 108 schizophrenia-associated genetic loci. *Nature* **511**, 421-427 (2014).

14. Autism Spectrum Disorders Working Group of The Psychiatric Genomics, C. Meta-analysis of GWAS of over 16,000 individuals with autism spectrum disorder highlights a novel locus at 10q24.32 and a significant overlap with schizophrenia. *Mol Autism* **8**, 21 (2017).

15. Mattheisen, M. et al. Genome-wide association study in obsessive-compulsive disorder: results from the OCGAS. *Mol Psychiatry* **20**, 337-344 (2015).

16. Pers, T.H., Timshel, P. &amp; Hirschhorn, J.N. SNPsnap: a Web-based tool for identification and annotation of matched SNPs. *Bioinformatics* **31**, 418-420 (2015).

17. Arnold, M., Raffler, J., Pfeufer, A., Suhre, K. &amp; Kastenmuller, G. SNiPA: an interactive, genetic variant-centered annotation browser. *Bioinformatics* **31**, 1334-1336 (2015).

18. Beagan, J.A. et al. Local Genome Topology Can Exhibit an Incompletely Rewired 3D-Folding State during Somatic Cell Reprogramming. *Cell Stem Cell* **18**, 611-624 (2016).

19. Gilgenast, T.G. & Phillips-Cremins, J.E. Systematic Evaluation of Statistical Methods for Identifying Looping Interactions in 5C Data. *Cell Syst* **8**, 197-211 e113 (2019).

20. Kim, J.H. et al. 5C-ID: Increased resolution Chromosome-Conformation-Capture-Carbon-Copy with in situ 3C and double alternating primer design. *Methods* **142**, 39-46 (2018).

21. Norton, H.K. et al. Detecting hierarchical genome folding with network modularity. *Nat Methods* **15**, 119-122 (2018).

22. Sun, J.H. et al. Disease-Associated Short Tandem Repeats Co-localize with Chromatin Domain Boundaries. *Cell* (2018).

23. Sauria, M.E., Phillips-Cremins, J.E., Corces, V.G. & Taylor, J. HiFive: a tool suite for easy and efficient HiC and 5C data analysis. *Genome Biol* **16**, 237 (2015).
